# A new panel of SNPs to assess thyroid carcinoma risk: a pilot study in a Brazilian admixture population

**DOI:** 10.1186/s12881-017-0502-8

**Published:** 2017-11-25

**Authors:** Isabelle C. C. dos Santos, Julieta Genre, Diego Marques, Ananília M. G. da Silva, Jéssica C. dos Santos, Jéssica N. G. de Araújo, Victor H. R. Duarte, Angel Carracedo, Maria Torres-Español, Gisele Bastos, Carlos C. de Oliveira Ramos, André D. Luchessi, Vivian N. Silbiger

**Affiliations:** 1 Department of Clinical Analysis and Toxicology of Federal University of Rio Grande do Norte, Rua General Gustavo Cordeiro de Farias s/n, CEP 59012-570, Natal, Rio Grande do Norte Brazil; 2 Health Sciences Posgraduation Programme, Federal University of Rio Grande do Norte, Natal, Brazil; 3 Grupo de Medicina Xenómica-CIBERER-Universidade de Santiago de Compostela. Fundación Pública Galega de Medicina Xenómica. Servicio Galego de Saúde, Santiago de Compostela, Spain; 4 Centro Nacional de Genotipado, PRB2- ISCIII. Universidade de Santiago de Compostela, Santiago de Compostela, Spain; 5 Department of Clinical Analysis and Toxicology of São Paulo University, São Paulo, SP Brazil; 6 Liga Norte Riograndense Contra o Câncer, Rio Grande do Norte, RN Brazil

**Keywords:** Thyroid cancer, Polymorphism, Predisposition

## Abstract

**Background:**

Thyroid cancer is a common malignant disease of the endocrine system with increasing incidence rates over the last few decades. In this study, we sought to analyze the possible association of 45 single nucleotide polymorphisms (SNPs) with thyroid cancer in a population from Rio Grande do Norte, Brazil.

**Methods:**

Based on histological analysis by a pathologist, 80 normal thyroid specimens of tissue adjacent to thyroid tumors were obtained from the biobank at the Laboratory of Pathology of Liga Norte Riograndense Contra o Câncer, Natal, RN. Patient samples were then genotyped using the MassARRAY platform (Sequenon, Inc) followed by statistical analysis employing the SNPassoc package in R program. The genotypic frequencies of all 45 SNPs obtained from the International HapMap Project database and based on data from the ancestral populations of European and African origin were used to compose the control study group.

**Results:**

In our study, the following 9 SNPs showed significant differences in their frequency when comparing the study and control groups: rs3744962, rs258107, rs1461855, rs4075022, rs9943744, rs4075570, rs2356508, rs17485896, and rs2651339. Furthermore, the SNPs rs374492 C/T and rs258107 C/T were associated with a relative risk for thyroid carcinoma of 3.78 (*p* = 6.27 × 10e^−5^) and 2.91 (*p* = 8.27 × 10e^−5^), respectively, after Bonferroni’s correction for multiple comparisons.

**Conclusions:**

These nine polymorphisms could be potential biomarkers of predisposition to thyroid carcinoma in the population from Rio Grande do Norte. However, complementary studies including a control group with samples obtained from healthy subjects in Rio Grande do Norte state, should be conducted to confirm these results.

**Electronic supplementary material:**

The online version of this article (10.1186/s12881-017-0502-8) contains supplementary material, which is available to authorized users.

## Background

Thyroid cancer (TC) is the most common endocrine malignancy, and its incidence rate has been increasing noticeably for decades [1]. Hence, if the observed trends are maintained, TC will replace colorectal cancer as the fourth leading cancer diagnosis in the United States by 2030 [2]. Such an increase is likely due to improved diagnosis, and largely or completely reflects the over diagnosis of indolent disease [3]. According to the Brazilian National Cancer Institute, about 6.960 new cases of TC were expected in Brazil in 2016 [4]. The state of Rio Grande do Norte, located in the northeast region of Brazil, presents the fourth highest estimated TC incidence rate in the country, which corresponds to 130 cases per 100,000 women and 30 cases per 100,000 men [4].

The thyroid gland is mainly composed of two cell types: follicular cells and parafollicular cells. Based on histological and clinical parameters, follicular cell-derived carcinomas, which correspond to more than 90% of thyroid carcinomas, are typically divided into well differentiated, poorly differentiated, and undifferentiated (or anaplastic) carcinomas. Well differentiated thyroid carcinomas (WDTC) are further subdivided histologically as papillary thyroid carcinoma and follicular thyroid carcinoma, which correspond to 80% and 10% of thyroid carcinomas, respectively [5]. Both these carcinomas can progress to poorly differentiated carcinoma or completely lose differentiation, giving rise to anaplastic carcinoma [6]. On the other hand, parafollicular cell-derived medullary carcinoma accounts for about 5% of thyroid carcinomas [5, 7].

The recent progress in understanding the molecular pathogenesis of TC has paved the way for developing more effective treatment strategies. This has mainly resulted from identification of numerous genetic and epigenetic changes including mutations, gene copy-number gain, gene translocation, and aberrant gene methylation, resulting in alterations of the signaling pathways involved in regulating cell proliferation and survival, such as the MAPK and PI3K–AKT pathways among others, which are reshaping TC medicine [8, 9]. In addition, it is widely accepted that genetic predisposition to WDTC is expected to have common low-penetrance and rare moderate-penetrance genetic variants interacting with each other and with the environment determining individual susceptibility [10].

Recent high-throughput genotyping (GWAS) studies sought to identify single nucleotide polymorphisms (SNPs) associated with increased risk of thyroid tumorigenesis and to better explain the role of genetic variations in predisposition to TC [10–22]. However, some studies have demonstrated contradictory results, probably due to genetic diversity both within and among populations. This observation raises the question of whether a new panel of SNPs could be used as potential predisposition markers for TC.

Therefore, this study aimed to investigate whether the selected SNPs could be associated with predisposition to TC in patients diagnosed with this neoplasia in the state of Rio Grande do Norte. Considering that the population of this region is characterized by a marked ethnic mixture, this study may significantly contribute to elucidating the molecular basis underlying both predisposition to TC and the effect of interbreed populations on SNP-based association studies.

## Methods

### Study group

In this retrospective study, 80 tumor formalin-fixed paraffin-embedded (FFPE) samples of tissue adjacent to thyroid tumors were selected from patients diagnosed with thyroid cancer at the Laboratory of Pathology, Hospital Liga Norte Riograndense Contra o Câncer (Natal, Brazil) between 2003 and 2011. Among these 80 cases, 77 had papillary thyroid carcinoma and 3 had follicular thyroid carcinoma, as confirmed based on histological analysis by a pathologist. Ten 20-μm sections were cut from blocks containing non-tumor tissue using a Rotary Microtome YD-335 (ANCAP, São Paulo, Brazil). Samples were placed in 1.5 mL microtubes at room temperature until processing. For each patient sample, a unique High Profile 818 disposable blade (Leica Microsystems GmbH, Wetzlar, Germany) was used to avoid contamination of genomic DNA.

The control group was obtained from the International HapMap project and consisted of genotype data from 180 healthy individuals: 90 with European ancestry and 90 with African ancestry (HapMap Genome Browse, release#24 Phase 1 & 2 - full dataset).

This research was approved by the Ethics Committee in Research from Hospital Liga Norte Riograndense Contra o Câncer (protocol number 558788).

### DNA extraction

FFPE tissue samples were deparaffinized with 2.4 mL of xylene (2 × 5 min at 14,000 rpm) and washed with 2.4 mL of 100% ethanol (2 × 5 min at 14,000 rpm). DNA was then isolated using the QIAamp® DNA FFPE Tissue Kit (QIAGEN, Valencia, EUA), according to the manufacturer’s instruction. DNA quantification was performed using the NanoDrop ND-1000 spectrophotometer. DNA samples were diluted to a final concentration of 20 ng/μL.

### SNP selection

A panel of 45 SNPs was selected through previous studies performed at the Genotyping National Center (CEGEN) in Santiago de Compostela – Spain (www.usc.es/cegen/), coordinated by Dr. Angel Carracedo, Professor of the Faculty of Medicine, University of Santiago de Compostela (USC) [10].

### Genotyping

SNP genotyping of the case group was performed using the MassARRAY SNP genotyping system (Agena Bioscience San Diego, EUA) according to manufacturer’s instructions at the National Genotyping Center (CEGEN), on a panel of 45 SNP assays. The primers for amplification and extension were designed using the Extend Primer Assay Design software v4. Sequenom iPLEX GOLD chemistry was used for locus-specific amplification, followed by a single-base primer extension reaction, which generated products of different masses that were quantitatively analyzed using MALDI-TOF mass spectrometry. The resulting data were analyzed using TyperAnalyzer software v 4, followed by manual inspection of the spectra by trained personnel [23]. All assays were performed in 384-well plates, including negative controls and a trio of Coriell samples (Na10830, Na10831, and Na12147) for quality control.

### Statistical analysis

Statistical tests were performed using the SNPassoc function in the R software v2.14.1 statistical package (R Development Core Team, 2006) [24]. For each SNP, associations were assessed by applying logistic regression to estimate the odds ratios (ORs) with 95% confidence intervals and *P* values. A P value <0.05 was considered statistically significant.

## Results

Eighty TC patients from Brazil were genotyped for 45 SNPs, to identify potential molecular markers of predisposition to this neoplasia. Among all the patients studied, 77 were diagnosed with papillary thyroid carcinoma and 3 with follicular thyroid carcinoma. The age of one patient was not determined, and the staging, location, and size of tumor were also not determined in some patients. Among the 80 patients, 61 lymph node metastases were not evaluated. These data are shown in Table 1.

**Table 1:** Clinical and pathological characteristics of patients enrolled in this study

Variables		N	%
Gender	Female	69	86%
	Male	11	14%
	Total	80	100%
Classification	Papillary	77	96%
	Follicular	3	4%
	Total	80	100%
Age (years)	< 45	38	48%
	≥ 45	41	52%
	Uninformed	1	1%
	Total	80	100%
TNM stage	I	48	61%
	II	1	1%
	III	29	37%
	IV	1	1%
	Uninformed	1	1%
	Total	79	100%
Tumor size (cm)	≤ 2	55	72%
	2 < size ≤4	18	23%
	> 4	4	5%
	Uninformed	3	4%
	Total	77	100%
Multicentricity	Yes	9	12,5%
	No	63	87,5%
	Uninformed	8	10%
	Total	72	100%
Extrathyroidal extension	Yes	49	61%
	No	31	39%
	Total	80	100%
Histological subtype	Classic	64	80%
	Follicular	11	14%
	Encapsulated follicular	3	4%
	Oxyphilic	2	2%
	Total	80	100%

The allelic frequencies of 45 SNPs were analyzed and the minimum cutoff value for missing genotypes was defined as 10%. The allelic composition, allele major frequency and the *p* value for the Hardy-Weinberg equilibrium (HWE) of each SNP are shown in Table 2. Of these 45 SNPs, 11 were excluded from the study because they were not in the HWE (*p* < 0.05). The allelic frequencies from the Brazilian population were compared with those available in the HapMap database for 180 individuals of European and African ancestry, which constituted the control group.

**Table 2:** SNPs evaluated according to Hardy-Weinberg equilibrium. The allele information were showed as Reference allele/Risk allele. P < 0.05 in bold. SNPs. Single nucleotide polymorphism; MAF: Minor Alleles frequency; HWE: Hardy-Weinberg Equilibrium. MAF YRI Minor Alleles frequency of population African; MAF EUR Minor Alleles frequency of European according with 1000 genomes project

SNPs	Allele	MAF	HWE	Missing Genotypes (%)	MAF YRI	MAF EUR
rs11749656	C/A	2.4	1.0000	3.1	–	5.0
rs1254167	G/C	8.7	1.0000	2.3	11.1	10.1
rs17026194	G/A	1.9	1.0002	1.2	5.5	–
rs17821714	G/A	3.7	1.0003	0.8	–	6.5
rs4245211	A/G	34.9	0.8885	5.8	40.7	26.7
rs6507639	T/G	7.1	1.0000	2.3	11.1	7,5
rs2910164	G/C	34.5	0.8899	1.9	–	–
rs6578493	A/C	19.9	0.8439	4.2	22.6	22.7
rs6825379	A/G	14.5	0.6189	3.1	30.5	5.0
rs664677	T/C	35.7	1.0000	4.2	19.4	44.4
rs10790373	C/T	27.8	0.7458	10	30.0	20.7
rs2651339	A/C	48.2	0.5273	4.6	44.4	48.4
rs566309	C/T	8.6	0.7043	1.5	11.1	9.6
rs2356508	C/A	8.7	1.0000	2.3	10.6	7.5
rs3744962	T/C	7.3	0.6285	2.3	0.9	8.5
rs17485896	T/C	22.7	0.4762	2.7	8.3	34.8
rs11856964	A/C	12.2	0.5525	1.9	14.3	13.1
rs1158257	C/T	46.9	0.7065	1.5	39.8	48.9
rs9943744	C/T	46.7	0.5285	2.3	28.2	42.9
rs7028661	G/A	31.0	0.3770	3.8	15.7	35.3
rs258107	C/T	27.9	0.4344	3.5	17.1	30.3
rs9993140	G/A	3.9	0.3222	2.3	15.2	1.5
rs31872	T/C	22.7	0.1938	8.5	8.8	33.8
rs11720059	G/A	6.0	0.2120	3.5	–	9.0
rs949908	T/A	47.6	0.1593	5.8	45.3	45.4
rs965513	G/A	29.1	0.2244	1.5	13.4	35.5
rs4075022	T/C	28.2	0.0827	5.8	6.0	43.4
rs12137541	G/A	22.4	0.0701	2.3	16.6	27.7
rs1801516	G/A	12.3	0.0869	1.2	–	18.1
rs1499008	G/A	37.2	0.1066	2.7	22.2	47.4
rs1461855	T/A	20.2	0.0799	1.9	45.8	8.0
rs2839582	T/C	40.7	0.0671	3.1	47.6	29.2
rs4075570	G/A	49.2	0.1263	5.0	39.3	38.8
rs7037324	G/A	26.5	0.0504	3.5	7.4	39.3
**rs6983267**	**G/T**	**31.0**	**0.0236**	**6.9**	**2.7**	**48.4**
**rs4698951**	**T/C**	**26.8**	**0.0103**	**1.2**	**37.9**	**6.0**
**rs17072086**	**T/C**	**15.9**	**0.0038**	**1.9**	**0.4**	**22.7**
**rs2284734**	**G/A**	**46.3**	**0.0027**	**1.2**	**20.8**	**33.8**
**rs2997312**	**G/A**	**28.1**	**0.0079**	**2.3**	**43.9**	**12.6**
**rs10912963**	**G/C**	**40.9**	**0.0059**	**4.6**	**17.5**	**44.9**
**rs12206214**	**G/A**	**28.8**	**0.0056**	**3.1**	**17.5**	**42.9**
**rs13447450**	**C/T**	**17.4**	**0.0007**	**1.5**	**0.9**	**37.8**
**rs162270**	**G/T**	**15.1**	**0.0002**	**2.7**	**–**	**–**
**rs9937860**	**T/G**	**15.1**	**0.0002**	**3.5**	**16.6**	**12.1**
**rs944289**	**C/T**	**38.1**	**0.00004**	**1.2**	**12.9**	**39.3**

The allele information were showed as Reference allele/Risk allele. *P* < 0.05 in bold. SNPs. Single nucleotide polymorphism; *MAF* Minor Alleles frequency, *HWE* Hardy-Weinberg Equilibrium. *MAF YRI* Minor Alleles frequency of population African; *MAF EUR* Minor Alleles frequency of European according with 1000 genomes project

The association analysis between patients and control groups for SNPs that showed a *p*-value greater than 0.05 for Hardy-Weinberg equilibrium is shown in Table 3 and Additional file 1: Table S1, which classify SNPs according to the inheritance model: dominant (AA/Ab + bb); recessive (AA + Ab/bb); over dominant (Ab/AA + bb) and co-dominant (AA/Ab/bb). As observed, 7 SNPs showed significant expression in the co-dominant model (rs1461855, rs2356508, rs258107, rs3744962, rs4075022, rs4075570, and rs9943744), 5 in the dominant model (rs1461855, rs258107, rs3744962, rs4075022, and rs9943744), 4 in the recessive model (rs1461855, rs2651339, rs4075570, and rs9943744) and 6 in the over dominant model (rs1461855, rs17485896, rs2356508, rs258107, rs3744962, and rs4075022). Most SNPs presented statistical significance under more than one model of inheritance. Furthermore, the SNPs rs374492 C/T and rs258107 C/T were associated with a relative risk for TC of 3.78 (*p* = 6.27 × 10e^−5^) and 2.91 (*p* = 8.27 × 10e^−5^), respectively after Bonferroni’s correction for multiple comparisons.

**Table 3:** Association analysis of thyroid carcinoma and SNPs under different genetic inheritance model. The SNPs rs258107 and rs3744962 remained significant after the correction of multiple Bonferroni test. *P* < 0.05 in bold. Dominant, AA/Ab + bb; Recessive, AA + Ab/bb; Overdominant, Ab/AA + bb; e Co-dominant, AA/Ab/bb; SNP,single nucleotide polymorphism

	Genetic Inheritance Model			
SNP	Codominant	Dominant	Recessive	Overdominant
**rs3744962**	**0.00004**	**0.00006**	0.08869	**0.00037**
**rs258107**	**0.00011**	**0.00002**	0.19808	**0.00038**
**rs9943744**	**0.00832**	**0.02203**	**0.00586**	0.74091
**rs1461855**	**0.01031**	**0.00866**	**0.02415**	0.09031
**rs4075022**	**0.01321**	**0.00572**	0.05205	0.11500
**rs2356508**	**0.01968**	0.19642	0.08636	0.05817
**rs4075570**	**0.02358**	**0.04191**	**0.01359**	0.65497
**rs2651339**	**0.03293**	0.83057	**0.01936**	**0.02445**
rs17821714	0.13175	–	–	–
**rs17485896**	0.13440	0.37340	0.14229	0.11092
rs7037324	0.23323	0.10739	0.95633	0.10064
rs6825379	0.27488	0.85283	0.18560	0.45379
rs949908	0.28475	0.21267	0.64497	0.12649
rs965513	0.32316	0.13310	0.56678	0.23804
rs2910164	0.39720	0.64797	0.17431	0.68458
rs1158257	0.42592	0.32289	0.25281	0.93547
rs566309	0.43874	0.24447	1	0.20038
rs12137541	0.44455	0.25309	0.89503	0.20454
rs17026194	0.45068	–	–	–
rs7028661	0.48507	0.24038	0.51989	0.43616
rs664677	0.51326	0.24855	0.67168	0.38747
rs1254167	0.55835	0.27937	1	0.38631
rs6578493	0.57504	0.92152	0.30054	0.74190
rs11856964	0.59493	0.35849	0.55739	0.42250
rs1801516	0.70738	–	–	–
rs11749656	0.71229	–	–	–
rs1499008	0.75451	0.69100	0.66191	0.46602
rs2839582	0.78512	0.60386	0.53001	0.99911
rs4245211	0.78865	0.80208	0.59695	0.55160
rs31872	0.81039	0.85747	0.51785	0.86577
rs11720059	0.84074	0.82070	0.55916	0.95422
rs10790373	0.89519	0.88354	0.70565	0.71643
rs6507639	1	0.93703	1	0.96945

**Fig. 1:**
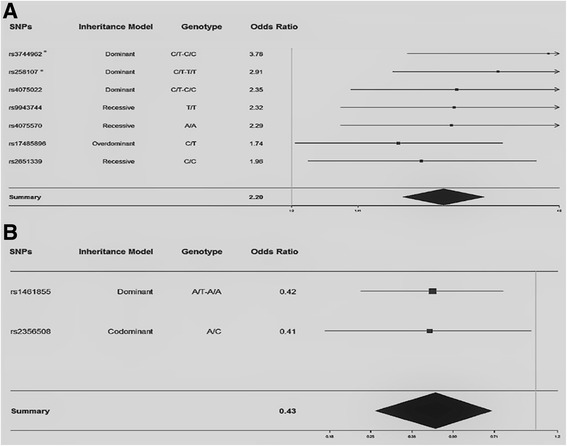
Associations between 9 SNPs and thyroid cancer susceptibility analyzed by the Forest plot. **a** All SNPs that show significant risk associated with thyroid cancer development. **b** All SNPs that show significant protection against thyroid cancer development. The summary represents a combination of SNPs that show risk or protection regarding cancer development

The SNPs rs258107 and rs3744962 remained significant after the correction of multiple Bonferroni test. P < 0.05 in bold. Dominant, AA/Ab + bb; Recessive, AA + Ab/bb; Overdominant, Ab/AA + bb; e Co-dominant, AA/Ab/bb; *SNP* single nucleotide polymorphism;

Figure 1 shows the results for logistic regression analysis of significant SNPs according to the model of inheritance, their respective odds ratios (OR), and *p*-values. Among the 9 SNPs analyzed (rs1461855, rs17485896, rs2356508, rs258107, rs2651339, rs3744962, rs4075022, rs4075570, and rs9943744), 7 showed OR values greater than 1, meaning that those alleles represent a risk for thyroid cancer development. In particular, the SNP rs3744962 showed the greatest risk with an OR value of 3.78. On the other hand, 2 SNPs (rs1461855 and rs2356508) were associated with a protective effect, with OR values less than 1.

## Discussion

The genetic ancestry profile of Brazilian population samples have been largely investigated in the last decades, mainly using uniparental inherited markers such mitochondrial DNA and Y-chromosome polymorphisms [25–29], as well as autosomal STR markers [30, 31]. In addition, some studies used ancestry informative markers such as SNPs and insertion/deletion polymorphisms have been used to estimate genetic ancestry [32–36].

All these previous studies conclude that the genetic ancestry of the Brazilian population is widely heterogeneous and is characterized by extensive admixture from three different ancestral roots: Amerindians, Europeans, and Africans. Furthermore, the composition of the Brazilian population ancestry follows a clear trend related to historical facts [37, 38]. European colonization of Brazil showed successive migration waves, starting with the arrival of around 500,000 Portuguese men between the years 1500 and 1800, when they met the local Amerindian population. Portuguese-Amerindian admixture began soon after arrival of the first colonizers. Simultaneously, about 4 million Africans were compulsorily introduced into Brazil as slaves until the mid-eighteenth century, providing the second major ethnic contribution to the Brazilian population. Later, other waves of immigration to Brazil occurred, mainly from Italy, Portugal, Spain, Germany, Syria, Lebanon, and Japan, adding even more complexity to the already multi-ethnic highly admixed Brazilian population [37, 38].

Brazil is the fifth largest country in the world and is divided into five geographical regions. In all these regions, the European ancestry is predominant with proportions ranging from 60.6% in the Northeast to 77.7% in the South [34, 39–42]. The African and Amerindian contributions to the population genetic background vary according to the region. The African ancestry proportion was the second largest in the Northeast (30.3%), followed in decreasing order by the Southeast, South, and North. On the other hand, populations in the North consist of a significant proportion of Amerindian ancestry, while remaining relatively uniform in other regions. In particular, the northeast region of Brazil where the state of Rio Grande do Norte is located, presents the highest African ancestry contribution in the country ranging from 18.6 to 56.8% [34, 39–43].

The heterogeneity and admixture of Brazilians constitutes an important issue to be considered in genetic association studies [34]. Besides, it has important clinical implications for the design and interpretation of clinical trials and genetic counseling. Because of its heterogeneous Amerindian, European, and African ancestral roots, the Brazilian population has been considered an important model for population genetics. In this context, our work aimed to genotype and assess the allele frequency of 45 SNPs to identify potential molecular markers of predisposition to TC in the population of Rio Grande do Norte, compared to the frequency of these alleles in European and African populations.

An extensive review of literature showed that considerable progress towards understanding complex diseases has been made in recent years due to development of high-throughput genotyping technologies [44]. Furthermore, several GWAS studies have identified SNPs associated with many complex diseases or traits. In the particular case of TC, genetic risk loci are under characterization in terms of allelic variants or genes responsible for association with the disease, as well as the affected biological pathway and the main cell type driving its pathology [45].

In this regard, a growing number of studies have identified several SNPs associated with TC risk [10–17, 19, 20, 22, 46–49]. In the study by Gudmundsson and collaborators [46], a GWAS analysis was performed in a population from Iceland and showed a strong association of SNPs rs965513 and rs944289 with papillary and follicular thyroid cancer. Furthermore, rs944289 and rs965513 correspond to gene variants of Thyroid transcription factor 1 and 2, respectively, and both TC risk alleles were associated with low concentrations of thyroid stimulating hormone, and SNP rs965513 was associated with low thyroxin concentration and high triiodothyronine concentration [46]. Later, the same group found that rs966423, rs2439302, and rs116909374 variants were also associated with TC [11].

The association of rs944289 and rs965513 with TC risk was also shown in genetic studies conducted in Asian populations [14, 50, 51]. Additionally, Wang and collaborators [48] observed that SNPs rs966423 and rs2439302, previously reported to be associated with TC in European populations, were confirmed as risk factors in the Chinese population.

Similarly, another study has demonstrated that SNPs rs2910164, rs965513, rs1867277, rs6983267, and rs944289 showed a significant association with TC risk in patients from the United Kingdom [12]. Furthermore, according to Jendrzejewski [52], the SNP rs944289 can predispose to papillary thyroid carcinoma through deregulation of PTCSC3 expression, which acts as a tumor suppressor.

A correlation study between Forkhead box E1 (FOXE1) gene variants rs894673, rs1867277, and rs3758249, and histopathological features of TC, suggested that FOXE1 variations generate a higher risk for poor histopathological features in papillary thyroid carcinoma [17]. In addition, Mancikova et al., [10] showed an association between SNPs rs7028661 and rs7037324, located near the FOXE1 locus, and TC risk. Moreover, the rare alleles of three SNPs (rs2997312, rs10788123 and rs1254167) showed suggestive evidence of association with higher risk for the disease. On the other hand, the SNP rs4075570 conferred protection in the series studied.

Finally, Lidral [15] showed that rs7850258 G allele associated with cleft lip, cleft palate, and hypothyroidism, has significantly greater enhancer activity than the allele associated with thyroid cancer (A); and Wokolorczyk et al., [47] showed that the SNP rs6983267 could be a good candidate multi-cancer susceptibility marker, once this SNP is associated with a wide range of cancers affecting the colon, prostate, breast, bladder, larynx, lung, kidney, and the thyroid [47]. These are some examples of the rapidly increasing number of studies focused on identifying genetic variants associated with TC predisposition and development.

An important challenge faced by molecular epidemiological association studies of candidate disease-susceptibility genes is to define variants that are functionally implicated in the disease. In addition to identifying polymorphic genetic variants and their possible association with diseases, it is necessary to understand the functional relevance of these SNPs. This is particularly urgent because the amount of genomic information that is available greatly exceeds the information about the function of variants that are presented in human disease studies [53]. Importantly, most of the recent genetic studies only establish the statistical associations of genetic markers and the disease, without supporting evidence of functional relevance. In context of predisposition to TC, new light is given on the molecular mechanisms of genetic variants through advances in molecular technologies.

As an example, the association of polymorphisms in DNA repair genes XRCC1 (rs25487, rs1799782) and XRCC3 (rs861539) with thyroid cancer risk and progression can be considered. As Yan and collaborators showed, the XRCC1 variant can interact with the XRCC3 variant to significantly increase differentiated thyroid carcinoma (DTC) susceptibility [22]. Similarly, a study to assess the role of polymorphisms in the LEP (rs7799039 and rs2167270) and LEPR (rs1137101 and rs1137100) genes in DTC susceptibility and their effect on leptin levels showed that LEP polymorphisms modify serum leptin concentrations in patients with DTC. Furthermore, the polymorphisms rs7799039 and rs1137101 increase the risk of DTC development, though they do not correlate with tumor aggressiveness [16]. As another example, supporting evidence of functional relevance was presented by Ceolin and collaborators [19], who evaluated the frequency of RET (REarranged during Transfection) proto-oncogene 3’UTR variants (rs76759170 and rs3026785) in Medullary Thyroid Carcinoma (MTC) patients. In silico analysis indicated that these variants might affect the secondary structure of RET mRNA, suggesting that they might play a role in the posttranscriptional control of RET transcripts.

In our study for molecular markers of predisposition to TC, we assessed the allele and genotype frequency of 45 SNPs, including four of the aforementioned SNPs. We observed that SNPs rs2910164 and rs965513, despite being in HWE, did not show associated risk with TC for the Brazilian population. Furthermore, SNPs rs6983267 and rs944289 were not in Hardy-Weinberg equilibrium in our studied groups. As observed, the frequencies of these particular SNPs differ from those observed for European, American and Asian populations, where some of these markers were associated with an increased risk of developing TC. These results reinforce the concept that the genetic constitution of a population, and the contribution of ancestral roots, is an important and influencing parameter in genetic association studies.

Other studies evaluated potential markers for predisposition to thyroid cancer in larger sample sizes [10, 46]. These studies were performed in European populations, but these kinds of studies were not previously performed in a Brazilian population. Therefore, this pilot study presents a good opportunity to examine the feasibility of our approach, exploring a novel panel of SNPs that could potentially predict TC risk. We have thus first investigated these SNPs in a pilot study with a plan to validate our findings in a larger sample in the future. It is critical to improve both our knowledge of thyroid cancer risk factors and our knowledge of markers that predict aggressive disease in order to reduce disease incidence and unnecessary treatments that result in undesirable side effects with long-term financial and clinical impacts.

## Conclusions

Of a total of 35 SNPs that were found in HWE in the Brazilian population, logistic regression analysis of 7 SNPs showed statistical significance when evaluated according to the genetic model of inheritance. For the first time, our results suggest that a new panel constituted of SNPs rs3744962, rs258107, rs4075022, rs9943744, rs4075570, rs17485896, and rs2651339 could represent predisposition to TC development in the population of Rio Grande do Norte. Furthermore, they may be considered suitable molecular markers for early diagnosis of the disease. These SNPs could be useful models to predict risk and for genetic counseling in clinical practice, thus avoiding invasive methods of analysis and often inconclusive diagnoses. In addition, SNPs rs1461855 and rs2356508 might possibly be associated with a protective effect against TC development. Nevertheless, complementary studies with a larger patient population as well as a control group composed of samples from the state of Rio Grande do Norte should be conducted to confirm these results.

## Additional file

Additional file 1: The logistic regression analyses between case-control and SNPs polymorphism. (DOCX 129 kb)

## Abbreviations

DTC: Differentiated Thyroid Carcinoma
FFPE: Formalin-fixed paraffin-embedded
FOXE1: Forkhead box E1
GWAS: High-throughput genotyping
HWE: Hardy-Weinberg equilibrium
MAF: Allele Minor Frequency
MTC: Medullary Thyroid Carcinoma
RET: REarranged during Transfection
SNPs: Single-nucleotide polymorphisms
TC: Thyroid cancer
WDTC: Well differentiated thyroid carcinomas
