# Supplementary material for: A new panel of SNPs to assess thyroid carcinoma risk: a pilot study in a Brazilian admixture population
Source: BMC Med Genet. 2017 Nov 25;18:140. doi: 10.1186/s12881-017-0502-8 (PMC5702224; doi:10.1186/s12881-017-0502-8)
Supplement: Additional file 1: — The logistic regression analyses between case-control and SNPs polymorphism. (DOCX 129 kb) [file 12881_2017_502_MOESM1_ESM.docx]

| **Supplementary Table 1 - The logistic regression analyses between case-control and SNPs polymorphism.** | | | | | | | | | |
| --- | --- | --- | --- | --- | --- | --- | --- | --- | --- |
| SNP | Control | % | Case | % | OR | lower | upper | p-value | AIC |
| rs10790373 |  |  |  |  |  |  |  |  |  |
| Codominant |  |  |  |  |  |  |  |  |  |
| C/C | 82 | 52.2 | 41 | 53.2 | 1 | [Ref] | | 0.8952 | 302.3 |
| C/T | 63 | 40.1 | 29 | 37.7 | 0.92 | 0.52 | 1.64 |  |  |
| T/T | 12 | 7.6 | 7 | 9.1 | 1.17 | 0.43 | 3.19 |  |  |
| Dominant |  |  |  |  |  |  |  |  |  |
| C/C | 82 | 52.2 | 41 | 53.2 | 1 | [Ref] | | 0.8835 | 300.5 |
| C/T-T/T | 75 | 47.8 | 36 | 46.8 | 0.96 | 0.56 | 1.66 |  |  |
| Recessive |  |  |  |  |  |  |  |  |  |
| C/C-C/T | 145 | 92.4 | 70 | 90.9 | 1] | [Ref] | | 0.7057 | 300.3 |
| T/T | 12 | 7.6 | 7 | 9.1 | 1.21 | 0.46 | 3.2 |  |  |
| Overdominant |  |  |  |  |  | [Ref] | |  |  |
| C/C-T/T | 94 | 59.9 | 48 | 62.3 | 1 |  | | 0.7164 | 300.4 |
| C/T | 63 | 40.1 | 29 | 37.7 | 0.9 | 0.51 | 1.58 |  |  |
| log-Additive |  |  |  |  |  |  |  |  |  |
| 0,1,2 | 157 | 67.1 | 77 | 32.9 | 1.01 | 0.66 | 1.55 | 0.9615 | 300.5 |
| SNP | Control | % | Case | % | OR | lower | upper | p-value | AIC |
| rs10912963 |  |  |  |  |  |  |  |  |  |
| Codominant |  |  |  |  |  |  |  |  |  |
| G/G | 79 | 45.4 | 18 | 24.3 | 1 [Ref] |  |  | 0.005339 | 297.8 |
| C/G | 64 | 36.8 | 35 | 47.3 | 2.4 | 1.24 | 4.63 |  |  |
| C/C | 31 | 17.8 | 21 | 28.4 | 2.97 | 1.4 | 6.32 |  |  |
| Dominant |  |  |  |  |  |  |  |  |  |
| G/G | 79 | 45.4 | 18 | 24.3 | 1 [Ref] |  |  | 0.001485 | 296.2 |
| C/G-C/C | 95 | 54.6 | 56 | 75.7 | 2.59 | 1.41 | 4.76 |  |  |
| Recessive |  |  |  |  |  |  |  |  |  |
| G/G-C/G | 143 | 82.2 | 53 | 71.6 | 1 [Ref] |  |  | 0.066833 | 302.9 |
| C/C | 31 | 17.8 | 21 | 28.4 | 1.83 | 0.97 | 3.46 |  |  |
| Overdominant |  |  |  |  |  |  |  |  |  |
| G/G-C/C | 110 | 63.2 | 39 | 52.7 | 1 [Ref] |  |  | 0.123454 | 303.9 |
| C/G | 64 | 36.8 | 35 | 47.3 | 1.54 | 0.89 | 2.68 |  |  |
| log-Additive |  |  |  |  |  |  |  |  |  |
| 0,1,2 | 174 | 70.2 | 74 | 29.8 | 1.75 | 1.21 | 2.52 | 0.002526 | 297.2 |
| SNP | Control | % | Case | % | OR | lower | upper | p-value | AIC |
| rs1158257 |  |  |  |  |  |  |  |  |  |
| Codominant |  |  |  |  |  |  |  |  |  |
| C/C | 55 | 30.7 | 19 | 24.7 | 1 [Ref] |  |  | 0.4259 | 317.4 |
| C/T | 87 | 48.6 | 37 | 48.1 | 1.23 | 0.64 | 2.35 |  |  |
| T/T | 37 | 20.7 | 21 | 27.3 | 1.64 | 0.78 | 3.47 |  |  |
| Dominant |  |  |  |  |  |  |  |  |  |
| C/C | 55 | 30.7 | 19 | 24.7 | 1 [Ref] |  |  | 0.3229 | 316.1 |
| C/T-T/T | 124 | 69.3 | 58 | 75.3 | 1.35 | 0.74 | 2.49 |  |  |
| Recessive |  |  |  |  |  |  |  |  |  |
| C/C-C/T | 142 | 79.3 | 56 | 72.7 | 1 [Ref] |  |  | 0.2528 | 315.8 |
| T/T | 37 | 20.7 | 21 | 27.3 | 1.44 | 0.78 | 2.67 |  |  |
| Overdominant |  |  |  |  |  |  |  |  |  |
| C/C-T/T | 92 | 51.4 | 40 | 51.9 | 1 [Ref] |  |  | 0.9355 | 317.1 |
| C/T | 87 | 48.6 | 37 | 48.1 | 0.98 | 0.57 | 1.67 |  |  |
| log-Additive |  |  |  |  |  |  |  |  |  |
| 0,1,2 | 179 | 69.9 | 77 | 30.1 | 1.28 | 0.88 | 1.86 | 0.1942 | 315.4 |
| SNP | Control | % | Case | % | OR | lower | upper | p-value | AIC |
| rs11720059 |  |  |  |  |  |  |  |  |  |
| Codominant |  |  |  |  |  |  |  |  |  |
| G/G | 156 | 89.1 | 67 | 88.2 | 1 [Ref] |  |  | 0.8407 | 313.5 |
| A/G | 18 | 10.3 | 8 | 10.5 | 1.03 | 0.43 | 2.5 |  |  |
| A/A | 1 | 0.6 | 1 | 1.3 | 2.33 | 0.14 | 37.78 |  |  |
| Dominant |  |  |  |  |  |  |  |  |  |
| G/G | 156 | 89.1 | 67 | 88.2 | 1 [Ref] |  |  | 0.8207 | 311.8 |
| A/G-A/A | 19 | 10.9 | 9 | 11.8 | 1.1 | 0.47 | 2.56 |  |  |
| Recessive |  |  |  |  |  |  |  |  |  |
| G/G-A/G | 174 | 99.4 | 75 | 98.7 | 1 [Ref] |  |  | 0.5592 | 311.5 |
| A/A | 1 | 0.6 | 1 | 1.3 | 2.32 | 0.14 | 37.58 |  |  |
| Overdominant |  |  |  |  |  |  |  |  |  |
| G/G-A/A | 157 | 89.7 | 68 | 89.5 | 1 [Ref] |  |  | 0.9542 | 311.8 |
| A/G | 18 | 10.3 | 8 | 10.5 | 1.03 | 0.43 | 2.47 |  |  |
| log-Additive |  |  |  |  |  |  |  |  |  |
| 0,1,2 | 175 | 69.7 | 76 | 30.3 | 1.15 | 0.54 | 2.44 | 0.72 | 311.7 |
| SNP | Control | % | Case | % | OR | lower | upper | p-value | AIC |
| rs11749656 |  |  |  |  |  |  |  |  |  |
| Codominant |  |  |  |  |  |  |  |  |  |
| C/C | 172 | 95.6 | 68 | 94.4 | 1 [Ref] |  |  | 0.7123 | 305.4 |
| A/C | 8 | 4.4 | 4 | 5.6 | 1.26 | 0.37 | 4.34 |  |  |
| log-Additive |  |  |  |  |  |  |  |  |  |
| 0,1,2 | 180 | 71.4 | 72 | 28.6 | 1.26 | 0.37 | 4.34 | 305.4 |  |
| SNP | Control | % | Case | % | OR | lower | upper | p-value | AIC |
| rs11856964 |  |  |  |  |  |  |  |  |  |
| Codominant |  |  |  |  |  |  |  |  |  |
| A/A | 139 | 78.1 | 56 | 72.7 | 1 [Ref] |  |  | 0.5949 | 317.3 |
| A/C | 38 | 21.3 | 20 | 26 | 1.31 | 0.7 | 2.44 |  |  |
| C/C | 1 | 0.6 | 1 | 1.3 | 2.48 | 0.15 | 40.38 |  |  |
| Dominant |  |  |  |  |  |  |  |  |  |
| A/A | 139 | 78.1 | 56 | 72.7 | 1 [Ref] |  |  | 0.3585 | 315.5 |
| A/C-C/C | 39 | 21.9 | 21 | 27.3 | 1.34 | 0.72 | 2.47 |  |  |
| Recessive |  |  |  |  |  |  |  |  |  |
| A/A-A/C | 177 | 99.4 | 76 | 98.7 | 1 [Ref] |  |  | 0.5574 | 316 |
| C/C | 1 | 0.6 | 1 | 1.3 | 2.33 | 0.14 | 37.72 |  |  |
| Overdominant |  |  |  |  |  |  |  |  |  |
| A/A-C/C | 140 | 78.7 | 57 | 74 | 1 [Ref] |  |  | 0.4225 | 315.7 |
| A/C | 38 | 21.3 | 20 | 26 | 1.29 | 0.69 | 2.41 |  |  |
| log-Additive |  |  |  |  |  |  |  |  |  |
| 0,1,2 | 178 | 69.8 | 77 | 30.2 | 1.34 | 0.75 | 2.4 | 0.3225 | 315.4 |
| SNP | Control | % | Case | % | OR | lower | upper | p-value | AIC |
| rs12137541 |  |  |  |  |  |  |  |  |  |
| Codominant |  |  |  |  |  |  |  |  |  |
| G/G | 116 | 64.4 | 42 | 56.8 | 1 [Ref] |  |  | 0.4446 | 310.9 |
| A/G | 51 | 28.3 | 27 | 36.5 | 1.46 | 0.81 | 2.62 |  |  |
| A/A | 13 | 7.2 | 5 | 6.8 | 1.06 | 0.36 | 3.16 |  |  |
| Dominant |  |  |  |  |  |  |  |  |  |
| G/G | 116 | 64.4 | 42 | 56.8 | 1 [Ref] |  |  | 0.2531 | 309.2 |
| A/G-A/A | 64 | 35.6 | 32 | 43.2 | 1.38 | 0.8 | 2.4 |  |  |
| Recessive |  |  |  |  |  |  |  |  |  |
| G/G-A/G | 167 | 92.8 | 69 | 93.2 | 1 [Ref] |  |  | 0.895 | 310.5 |
| A/A | 13 | 7.2 | 5 | 6.8 | 0.93 | 0.32 | 2.71 |  |  |
| Overdominant |  |  |  |  |  |  |  |  |  |
| G/G-A/A | 129 | 71.7 | 47 | 63.5 | 1 [Ref] |  |  | 0.2045 | 308.9 |
| A/G | 51 | 28.3 | 27 | 36.5 | 1.45 | 0.82 | 2.58 |  |  |
| log-Additive |  |  |  |  |  |  |  |  |  |
| 0,1,2 | 180 | 70.9 | 74 | 29.1 | 1.2 | 0.78 | 1.83 | 0.4054 | 309.8 |
| SNP | Control | % | Case | % | OR | lower | upper | p-value | AIC |
| rs12206214 |  |  |  |  |  |  |  |  |  |
| Codominant |  |  |  |  |  |  |  |  |  |
| G/G | 107 | 59.4 | 30 | 41.7 | 1 [Ref] |  |  | 0.015643 | 299.2 |
| A/G | 57 | 31.7 | 28 | 38.9 | 1.75 | 0.95 | 3.22 |  |  |
| A/A | 16 | 8.9 | 14 | 19.4 | 3.12 | 1.37 | 7.11 |  |  |
| Dominant |  |  |  |  |  |  |  |  |  |
| G/G | 107 | 59.4 | 30 | 41.7 | 1 [Ref] |  |  | 0.010505 | 299 |
| A/G-A/A | 73 | 40.6 | 42 | 58.3 | 2.05 | 1.18 | 3.57 |  |  |
| Recessive |  |  |  |  |  |  |  |  |  |
| G/G-A/G | 164 | 91.1 | 58 | 80.6 | 1 [Ref] |  |  | 0.024608 | 300.5 |
| A/A | 16 | 8.9 | 14 | 19.4 | 2.47 | 1.14 | 5.38 |  |  |
| Overdominant |  |  |  |  |  |  |  |  |  |
| G/G-A/A | 123 | 68.3 | 44 | 61.1 | 1 [Ref] |  |  | 0.276429 | 304.3 |
| A/G | 57 | 31.7 | 28 | 38.9 | 1.37 | 0.78 | 2.42 |  |  |
| log-Additive |  |  |  |  |  |  |  |  |  |
| 0,1,2 | 180 | 71.4 | 72 | 28.6 | 1.76 | 1.2 | 2.59 | 0.003932 | 297.2 |
| SNP | Control | % | Case | % | OR | lower | upper | p-value | AIC |
| rs1254167 |  |  |  |  |  |  |  |  |  |
| Codominant |  |  |  |  |  |  |  |  |  |
| G/G | 144 | 81.8 | 68 | 87.2 | 1 [Ref] |  |  | - | 317 |
| C/G | 30 | 17 | 10 | 12.8 | 0.71 | 0.33 | 1.53 |  |  |
| C/C | 2 | 1.1 | 0 | 0 | 0 | 0 |  |  |  |
| Dominant |  |  |  |  |  |  |  |  |  |
| G/G | 144 | 81.8 | 68 | 87.2 | 1 [Ref] |  |  | 0.2794 | 316.1 |
| C/G-C/C | 32 | 18.2 | 10 | 12.8 | 0.66 | 0.31 | 1.42 |  |  |
| Recessive |  |  |  |  |  |  |  |  |  |
| G/G-C/G | 174 | 98.9 | 78 | 100 | 1 [Ref] |  |  | - | 315.8 |
| C/C | 2 | 1.1 | 0 | 0 | 0 | 0 |  |  |  |
| Overdominant |  |  |  |  |  |  |  |  |  |
| G/G-C/C | 146 | 83 | 68 | 87.2 | 1 [Ref] |  |  | 0.3863 | 316.6 |
| C/G | 30 | 17 | 10 | 12.8 | 0.72 | 0.33 | 1.55 |  |  |
| log-Additive |  |  |  |  |  |  |  |  |  |
| 0,1,2 | 176 | 69.3 | 78 | 30.7 | 0.64 | 0.31 | 1.33 | 0.5584 | 315.8 |
| SNP | Control | % | Case | % | OR | lower | upper | p-value | AIC |
| rs13447450 |  |  |  |  |  |  |  |  |  |
| Codominant |  |  |  |  |  |  |  |  |  |
| C/C | 131 | 72.8 | 52 | 68.4 | 1 [Ref] |  |  | 0.08761 | 312.5 |
| C/T | 35 | 19.4 | 22 | 28.9 | 1.58 | 0.85 | 2.95 |  |  |
| T/T | 14 | 7.8 | 2 | 2.6 | 0.36 | 0.08 | 1.64 |  |  |
| Dominant |  |  |  |  |  |  |  |  |  |
| C/C | 131 | 72.8 | 52 | 68.4 | 1 [Ref] |  |  | 0.48307 | 314.9 |
| C/T-T/T | 49 | 27.2 | 24 | 31.6 | 1.23 | 0.69 | 2.21 |  |  |
| Recessive |  |  |  |  |  |  |  |  |  |
| C/C-C/T | 166 | 92.2 | 74 | 97.4 | 1 [Ref] |  |  | 0.09349 | 312.6 |
| T/T | 14 | 7.8 | 2 | 2.6 | 0.32 | 0.07 | 1.45 |  |  |
| Overdominant |  |  |  |  |  |  |  |  |  |
| C/C-T/T | 145 | 80.6 | 54 | 71.1 | 1 [Ref] |  |  | 0.10066 | 312.7 |
| C/T | 35 | 19.4 | 22 | 28.9 | 1.69 | 0.91 | 3.13 |  |  |
| log-Additive |  |  |  |  |  |  |  |  |  |
| 0,1,2 | 180 | 70.3 | 76 | 29.7 | 0.98 | 0.62 | 1.54 | 0.92238 | 315.4 |
| SNP | Control | % | Case | % | OR | lower | upper | p-value | AIC |
| rs1461855 |  |  |  |  |  |  |  |  |  |
| Codominant |  |  |  |  |  |  |  |  |  |
| T/T | 109 | 60.6 | 58 | 77.3 | 1 [Ref] |  |  | 0.010318 | 305.8 |
| A/T | 57 | 31.7 | 16 | 21.3 | 0.53 | 0.28 | 1 |  |  |
| A/A | 14 | 7.8 | 1 | 1.3 | 0.13 | 0.02 | 1.05 |  |  |
| Dominant |  |  |  |  |  |  |  |  |  |
| T/T | 109 | 60.6 | 58 | 77.3 | 1 [Ref] |  |  | 0.008665 | 306.1 |
| A/T-A/A | 71 | 39.4 | 17 | 22.7 | 0.45 | 0.24 | 0.83 |  |  |
| Recessive |  |  |  |  |  |  |  |  |  |
| T/T-A/T | 166 | 92.2 | 74 | 98.7 | 1 [Ref] |  |  | 0.024151 | 307.9 |
| A/A | 14 | 7.8 | 1 | 1.3 | 0.16 | 0.02 | 1.24 |  |  |
| Overdominant |  |  |  |  |  |  |  |  |  |
| T/T-A/A | 123 | 68.3 | 59 | 78.7 | 1 [Ref] |  |  | 0.09032 | 310.1 |
| A/T | 57 | 31.7 | 16 | 21.3 | 0.59 | 0.31 | 1.1 |  |  |
| log-Additive |  |  |  |  |  |  |  |  |  |
| 0,1,2 | 180 | 70.6 | 75 | 29.4 | 0.47 | 0.27 | 0.8 | 0.00313 | 304.2 |
| SNP | Control | % | Case | % | OR | lower | upper | p-value | AIC |
| rs1499008 |  |  |  |  |  |  |  |  |  |
| Codominant |  |  |  |  |  |  |  |  |  |
| G/G | 74 | 41.1 | 32 | 43.8 | 1 [Ref] |  |  | 0.7545 | 309.5 |
| A/G | 78 | 43.3 | 28 | 38.4 | 0.83 | 0.46 | 1.51 |  |  |
| A/A | 28 | 15.6 | 13 | 17.8 | 1.07 | 0.49 | 2.34 |  |  |
| Dominant |  |  |  |  |  |  |  |  |  |
| G/G | 74 | 41.1 | 32 | 43.8 | 1 [Ref] |  |  | 0.691 | 307.9 |
| A/G-A/A | 106 | 58.9 | 41 | 56.2 | 0.89 | 0.52 | 1.55 |  |  |
| Recessive |  |  |  |  |  |  |  |  |  |
| G/G-A/G | 152 | 84.4 | 60 | 82.2 | 1 [Ref] |  |  | 0.6619 | 307.8 |
| A/A | 28 | 15.6 | 13 | 17.8 | 1.18 | 0.57 | 2.42 |  |  |
| Overdominant |  |  |  |  |  |  |  |  |  |
| G/G-A/A | 102 | 56.7 | 45 | 61.6 | 1 [Ref] |  |  | 0.466 | 307.5 |
| A/G | 78 | 43.3 | 28 | 38.4 | 0.81 | 0.47 | 1.42 |  |  |
| log-Additive |  |  |  |  |  |  |  |  |  |
| 0,1,2 | 180 | 71.1 | 73 | 28.9 | 0.99 | 0.68 | 1.45 | 0.9622 | 308 |
| SNP | Control | % | Case | % | OR | lower | upper | p-value | AIC |
| rs162270 |  |  |  |  |  |  |  |  |  |
| Codominant |  |  |  |  |  |  |  |  |  |
| G/G | 150 | 83.3 | 56 | 76.7 | 1 [Ref] |  |  | 0.464 | 308.5 |
| G/T | 24 | 13.3 | 13 | 17.8 | 1.45 | 0.69 | 3.05 |  |  |
| T/T | 6 | 3.3 | 4 | 5.5 | 1.79 | 0.49 | 6.56 |  |  |
| Dominant |  |  |  |  |  |  |  |  |  |
| G/G | 150 | 83.3 | 56 | 76.7 | 1 [Ref] |  |  | 0.2276 | 306.6 |
| G/T-T/T | 30 | 16.7 | 17 | 23.3 | 1.52 | 0.78 | 2.96 |  |  |
| Recessive |  |  |  |  |  |  |  |  |  |
| G/G-G/T | 174 | 96.7 | 69 | 94.5 | 1 [Ref] |  |  | 0.4411 | 307.4 |
| T/T | 6 | 3.3 | 4 | 5.5 | 1.68 | 0.46 | 6.14 |  |  |
| Overdominant |  |  |  |  |  |  |  |  |  |
| G/G-T/T | 156 | 86.7 | 60 | 82.2 | 1 [Ref] |  |  | 0.369 | 307.2 |
| G/T | 24 | 13.3 | 13 | 17.8 | 1.41 | 0.67 | 2.95 |  |  |
| log-Additive |  |  |  |  |  |  |  |  |  |
| 0,1,2 | 180 | 71.1 | 73 | 28.9 | 1.38 | 0.83 | 2.31 | 0.2197 | 306.5 |
| SNP | Control | % | Case | % | OR | lower | upper | p-value | AIC |
| rs17026194 |  |  |  |  |  |  |  |  |  |
| Codominant |  |  |  |  |  |  |  |  |  |
| G/G | 171 | 95.5 | 76 | 97.4 | 1 [Ref] |  |  | 0.4507 | 318.9 |
| A/G | 8 | 4.5 | 2 | 2.6 | 0.56 | 0.12 | 2.71 |  |  |
| log-Additive |  |  |  |  |  |  |  |  |  |
| 0,1,2 | 179 | 69.6 | 78 | 30.4 | 0.56 | 0.12 | 2.71 | 318.9 |  |
| SNP | Control | % | Case | % | OR | lower | upper | p-value | AIC |
| rs17072086 |  |  |  |  |  |  |  |  |  |
| Codominant |  |  |  |  |  |  |  |  |  |
| T/T | 146 | 82 | 41 | 53.2 | 1 [Ref] |  |  | 2.84E-06 | 292.8 |
| C/T | 29 | 16.3 | 26 | 33.8 | 3.19 | 1.7 | 6.01 |  |  |
| C/C | 3 | 1.7 | 10 | 13 | 11.87 | 3.12 | 45.15 |  |  |
| Dominant |  |  |  |  |  |  |  |  |  |
| T/T | 146 | 82 | 41 | 53.2 | 1 [Ref] |  |  | 3.29E-06 | 294.7 |
| C/T-C/C | 32 | 18 | 36 | 46.8 | 4.01 | 2.22 | 7.22 |  |  |
| Recessive |  |  |  |  |  |  |  |  |  |
| T/T-C/T | 175 | 98.3 | 67 | 87 | 1 [Ref] |  |  | 3.47E-04 | 303.6 |
| C/C | 3 | 1.7 | 10 | 13 | 8.71 | 2.32 | 32.61 |  |  |
| Overdominant |  |  |  |  |  |  |  |  |  |
| T/T-C/C | 149 | 83.7 | 51 | 66.2 | 1 [Ref] |  |  | 2.43E-03 | 307.2 |
| C/T | 29 | 16.3 | 26 | 33.8 | 2.62 | 1.41 | 4.86 |  |  |
| log-Additive |  |  |  |  |  |  |  |  |  |
| 0,1,2 | 178 | 69.8 | 77 | 30.2 | 3.31 | 2.03 | 5.39 | 4.39E-07 | 290.9 |
| SNP | Control | % | Case | % | OR | lower | upper | p-value | AIC |
| rs17485896 |  |  |  |  |  |  |  |  |  |
| Codominant |  |  |  |  |  |  |  |  |  |
| T/T | 112 | 62.2 | 41 | 56.2 | 1 [Ref] |  |  | 0.1344 | 306 |
| C/T | 55 | 30.6 | 30 | 41.1 | 1.49 | 0.84 | 2.64 |  |  |
| C/C | 13 | 7.2 | 2 | 2.7 | 0.42 | 0.09 | 1.94 |  |  |
| Dominant |  |  |  |  |  |  |  |  |  |
| T/T | 112 | 62.2 | 41 | 56.2 | 1 [Ref] |  |  | 0.3734 | 307.2 |
| C/T-C/C | 68 | 37.8 | 32 | 43.8 | 1.29 | 0.74 | 2.23 |  |  |
| Recessive |  |  |  |  |  |  |  |  |  |
| T/T-C/T | 167 | 92.8 | 71 | 97.3 | 1 [Ref] |  |  | 0.1423 | 305.9 |
| C/C | 13 | 7.2 | 2 | 2.7 | 0.36 | 0.08 | 1.65 |  |  |
| Overdominant |  |  |  |  |  |  |  |  |  |
| T/T-C/C | 125 | 69.4 | 43 | 58.9 | 1 [Ref] |  |  | 0.1109 | 305.5 |
| C/T | 55 | 30.6 | 30 | 41.1 | 1.59 | 0.9 | 2.79 |  |  |
| log-Additive |  |  |  |  |  |  |  |  |  |
| 0,1,2 | 180 | 71.1 | 73 | 28.9 | 1.04 | 0.67 | 1.63 | 0.8515 | 308 |
| SNP | Control | % | Case | % | OR | lower | upper | p-value | AIC |
| rs17821714 |  |  |  |  |  |  |  |  |  |
| Codominant |  |  |  |  |  |  |  |  |  |
| G/G | 164 | 91.1 | 75 | 96.2 | 1 [Ref] |  |  | - | 317.9 |
| A/G | 16 | 8.9 | 3 | 3.8 | 0.41 | 0.12 | 1.45 |  |  |
| log-Additive |  |  |  |  |  |  |  |  |  |
| 0,1,2 | 180 | 69.8 | 78 | 30.2 | 0.41 | 0.12 | 1.45 | 0.945 | 317.9 |
| SNP | Control | % | Case | % | OR | lower | upper | p-value | AIC |
| rs1801516 |  |  |  |  |  |  |  |  |  |
| Codominant |  |  |  |  |  |  |  |  |  |
| G/G | 144 | 80 | 60 | 77.9 | 1 [Ref] |  |  | - | 317.7 |
| A/G | 36 | 20 | 17 | 22.1 | 1.13 | 0.59 | 2.17 |  |  |
| log-Additive |  |  |  |  |  |  |  |  |  |
| 0,1,2 | 180 | 70 | 77 | 30 | 1.13 | 0.59 | 2.17 | 1 | 317.7 |
| SNP | Control | % | Case | % | OR | lower | upper | p-value | AIC |
| rs2284734 |  |  |  |  |  |  |  |  |  |
| Codominant |  |  |  |  |  |  |  |  |  |
| G/G | 75 | 41.7 | 11 | 14.3 | 1 [Ref] |  |  | 4.63E-05 | 299.9 |
| A/G | 64 | 35.6 | 40 | 51.9 | 4.26 | 2.02 | 8.99 |  |  |
| A/A | 41 | 22.8 | 26 | 33.8 | 4.32 | 1.94 | 9.63 |  |  |
| Dominant |  |  |  |  |  |  |  |  |  |
| G/G | 75 | 41.7 | 11 | 14.3 | 1 [Ref] |  |  | 7.90E-06 | 297.9 |
| A/G-A/A | 105 | 58.3 | 66 | 85.7 | 4.29 | 2.12 | 8.66 |  |  |
| Recessive |  |  |  |  |  |  |  |  |  |
| G/G-A/G | 139 | 77.2 | 51 | 66.2 | 1 [Ref] |  |  | 7.01E-02 | 314.5 |
| A/A | 41 | 22.8 | 26 | 33.8 | 1.73 | 0.96 | 3.11 |  |  |
| Overdominant |  |  |  |  |  |  |  |  |  |
| G/G-A/A | 116 | 64.4 | 37 | 48.1 | 1 [Ref] |  |  | 1.47E-02 | 311.9 |
| A/G | 64 | 35.6 | 40 | 51.9 | 1.96 | 1.14 | 3.37 |  |  |
| log-Additive |  |  |  |  |  |  |  |  |  |
| 0,1,2 | 180 | 70 | 77 | 30 | 1.95 | 1.36 | 2.81 | 2.25E-04 | 304.2 |
| SNP | Control | % | Case | % | OR | lower | upper | p-value | AIC |
| rs2356508 |  |  |  |  |  |  |  |  |  |
| Codominant |  |  |  |  |  |  |  |  |  |
| C/C | 146 | 81.6 | 66 | 88 | 1 [Ref] |  |  | 0.01969 | 306 |
| A/C | 33 | 18.4 | 7 | 9.3 | 0.47 | 0.2 | 1.12 |  |  |
| A/A | 0 | 0 | 2 | 2.7 | 0 |  |  |  |  |
| Dominant |  |  |  |  |  |  |  |  |  |
| C/C | 146 | 81.6 | 66 | 88 | 1 [Ref] |  |  | 0.19643 | 310.6 |
| A/C-A/A | 33 | 18.4 | 9 | 12 | 0.6 | 0.27 | 1.33 |  |  |
| Recessive |  |  |  |  |  |  |  |  |  |
| C/C-A/C | 179 | 100 | 73 | 97.3 | 1 [Ref] |  |  | - | 307.3 |
| A/A | 0 | 0 | 2 | 2.7 | 0 |  |  |  |  |
| Overdominant |  |  |  |  |  |  |  |  |  |
| C/C-A/A | 146 | 81.6 | 68 | 90.7 | 1 [Ref] |  |  | 0.05817 | 308.7 |
| A/C | 33 | 18.4 | 7 | 9.3 | 0.46 | 0.19 | 1.08 |  |  |
| log-Additive |  |  |  |  |  |  |  |  |  |
| 0,1,2 | 179 | 70.5 | 75 | 29.5 | 0.78 | 0.38 | 1.59 | 0.01969 | 311.8 |
| SNP | Control | % | Case | % | OR | lower | upper | p-value | AIC |
| rs258107 |  |  |  |  |  |  |  |  |  |
| Codominant |  |  |  |  |  |  |  |  |  |
| C/C | 110 | 61.5 | 23 | 31.9 | 1 [Ref] |  |  | 1.12E-04 | 288.7 |
| C/T | 56 | 31.3 | 40 | 55.6 | 3.42 | 1.86 | 6.26 |  |  |
| T/T | 13 | 7.3 | 9 | 12.5 | 3.31 | 1.27 | 8.66 |  |  |
| Dominant |  |  |  |  |  |  |  |  |  |
| C/C | 110 | 61.5 | 23 | 31.9 | 1 [Ref] |  |  | 2.01E-05 | 286.7 |
| C/T-T/T | 69 | 38.5 | 49 | 68.1 | 3.4 | 1.9 | 6.06 |  |  |
| Recessive |  |  |  |  |  |  |  |  |  |
| C/C-C/T | 166 | 92.7 | 63 | 87.5 | 1 [Ref] |  |  | 1.98E-01 | 303.2 |
| T/T | 13 | 7.3 | 9 | 12.5 | 1.82 | 0.74 | 4.48 |  |  |
| Overdominant |  |  |  |  |  |  |  |  |  |
| C/C-T/T | 123 | 68.7 | 32 | 44.4 | 1 [Ref] |  |  | 3.88E-04 | 292.3 |
| C/T | 56 | 31.3 | 40 | 55.6 | 2.75 | 1.57 | 4.82 |  |  |
| log-Additive |  |  |  |  |  |  |  |  |  |
| 0,1,2 | 179 | 71.3 | 72 | 28.7 | 2.23 | 1.46 | 3.41 | 1.55E-04 | 290.5 |
| SNP | Control | % | Case | % | OR | lower | upper | p-value | AIC |
| rs2651339 |  |  |  |  |  |  |  |  |  |
| Codominant |  |  |  |  |  |  |  |  |  |
| A/A | 48 | 27.4 | 21 | 28.8 | 1 [Ref] |  |  | 0.03293 | 299.8 |
| A/C | 92 | 52.6 | 27 | 37 | 0.67 | 0.34 | 1.31 |  |  |
| C/C | 35 | 20 | 25 | 34.2 | 1.63 | 0.79 | 3.37 |  |  |
| Dominant |  |  |  |  |  |  |  |  |  |
| A/A | 48 | 27.4 | 21 | 28.8 | 1 [Ref] |  |  | 0.83058 | 304.5 |
| A/C-C/C | 127 | 72.6 | 52 | 71.2 | 0.94 | 0.51 | 1.72 |  |  |
| Recessive |  |  |  |  |  |  |  |  |  |
| A/A-A/C | 140 | 80 | 48 | 65.8 | 1 [Ref] |  |  | 0.01936 | 299.1 |
| C/C | 35 | 20 | 25 | 34.2 | 2.08 | 1.13 | 3.83 |  |  |
| Overdominant |  |  |  |  |  |  |  |  |  |
| A/A-C/C | 83 | 47.4 | 46 | 63 | 1 [Ref] |  |  | 0.02445 | 299.5 |
| A/C | 92 | 52.6 | 27 | 37 | 0.53 | 0.3 | 0.93 |  |  |
| log-Additive |  |  |  |  |  |  |  |  |  |
| 0,1,2 | 175 | 70.6 | 73 | 29.4 | 1.28 | 0.88 | 1.88 | 0.19809 | 302.9 |
| SNP | Control | % | Case | % | OR | lower | upper | p-value | AIC |
| rs2839582 |  |  |  |  |  |  |  |  |  |
| Codominant |  |  |  |  |  |  |  |  |  |
| T/T | 70 | 39.1 | 26 | 35.6 | 1 [Ref] |  |  | 0.7851 | 308.9 |
| C/T | 76 | 42.5 | 31 | 42.5 | 1.1 | 0.59 | 2.03 |  |  |
| C/C | 33 | 18.4 | 16 | 21.9 | 1.31 | 0.62 | 2.76 |  |  |
| Dominant |  |  |  |  |  |  |  |  |  |
| T/T | 70 | 39.1 | 26 | 35.6 | 1 [Ref] |  |  | 0.6039 | 307.1 |
| C/T-C/C | 109 | 60.9 | 47 | 64.4 | 1.16 | 0.66 | 2.04 |  |  |
| Recessive |  |  |  |  |  |  |  |  |  |
| T/T-C/T | 146 | 81.6 | 57 | 78.1 | 1 [Ref] |  |  | 0.53 | 306.9 |
| C/C | 33 | 18.4 | 16 | 21.9 | 1.24 | 0.63 | 2.43 |  |  |
| Overdominant |  |  |  |  |  |  |  |  |  |
| T/T-C/C | 103 | 57.5 | 42 | 57.5 | 1 [Ref] |  |  | 0.9991 | 307.3 |
| C/T | 76 | 42.5 | 31 | 42.5 | 1 | 0.58 | 1.73 |  |  |
| log-Additive |  |  |  |  |  |  |  |  |  |
| 0,1,2 | 179 | 71 | 73 | 29 | 1.14 | 0.79 | 1.64 | 0.4955 | 306.9 |
| SNP | Control | % | Case | % | OR | lower | upper | p-value | AIC |
| rs2910164 |  |  |  |  |  |  |  |  |  |
| Codominant |  |  |  |  |  |  |  |  |  |
| G/G | 76 | 42.2 | 34 | 45.3 | 1 [Ref] |  |  | 0.3972 | 313.1 |
| C/G | 79 | 43.9 | 35 | 46.7 | 0.99 | 0.56 | 1.75 |  |  |
| C/C | 25 | 13.9 | 6 | 8 | 0.54 | 0.2 | 1.43 |  |  |
| Dominant |  |  |  |  |  |  |  |  |  |
| G/G | 76 | 42.2 | 34 | 45.3 | 1 [Ref] |  |  | 0.648 | 312.7 |
| C/G-C/C | 104 | 57.8 | 41 | 54.7 | 0.88 | 0.51 | 1.52 |  |  |
| Recessive |  |  |  |  |  |  |  |  |  |
| G/G-C/G | 155 | 86.1 | 69 | 92 | 1 [Ref] |  |  | 0.1743 | 311.1 |
| C/C | 25 | 13.9 | 6 | 8 | 0.54 | 0.21 | 1.37 |  |  |
| Overdominant |  |  |  |  |  |  |  |  |  |
| G/G-C/C | 101 | 56.1 | 40 | 53.3 | 1 [Ref] |  |  | 0.6846 | 312.8 |
| C/G | 79 | 43.9 | 35 | 46.7 | 1.12 | 0.65 | 1.92 |  |  |
| log-Additive |  |  |  |  |  |  |  |  |  |
| 0,1,2 | 180 | 70.6 | 75 | 29.4 | 0.82 | 0.55 | 1.23 | 0.3302 | 312 |
| SNP | Control | % | Case | % | OR | lower | upper | p-value | AIC |
| rs2997312 |  |  |  |  |  |  |  |  |  |
| Codominant |  |  |  |  |  |  |  |  |  |
| G/G | 91 | 50.8 | 49 | 65.3 | 1 [Ref] |  |  | 0.003624 | 303 |
| A/G | 61 | 34.1 | 24 | 32 | 0.73 | 0.41 | 1.31 |  |  |
| A/A | 27 | 15.1 | 2 | 2.7 | 0.14 | 0.03 | 0.6 |  |  |
| Dominant |  |  |  |  |  |  |  |  |  |
| G/G | 91 | 50.8 | 49 | 65.3 | 1 [Ref] |  |  | 0.032871 | 307.7 |
| A/G-A/A | 88 | 49.2 | 26 | 34.7 | 0.55 | 0.31 | 0.96 |  |  |
| Recessive |  |  |  |  |  |  |  |  |  |
| G/G-A/G | 152 | 84.9 | 73 | 97.3 | 1 [Ref] |  |  | 0.001463 | 302.1 |
| A/A | 27 | 15.1 | 2 | 2.7 | 0.15 | 0.04 | 0.67 |  |  |
| Overdominant |  |  |  |  |  |  |  |  |  |
| G/G-A/A | 118 | 65.9 | 51 | 68 | 1 [Ref] |  |  | 0.748289 | 312.2 |
| A/G | 61 | 34.1 | 24 | 32 | 0.91 | 0.51 | 1.62 |  |  |
| log-Additive |  |  |  |  |  |  |  |  |  |
| 0,1,2 | 179 | 70.5 | 75 | 29.5 | 0.53 | 0.34 | 0.83 | 0.003361 | 303.7 |
| SNP | Control | % | Case | % | OR | lower | upper | p-value | AIC |
| rs31872 |  |  |  |  |  |  |  |  |  |
| Codominant |  |  |  |  |  |  |  |  |  |
| T/T | 111 | 61.7 | 35 | 60.3 | 1 [Ref] |  |  | 0.8104 | 269.9 |
| C/T | 58 | 32.2 | 18 | 31 | 0.98 | 0.51 | 1.89 |  |  |
| C/C | 11 | 6.1 | 5 | 8.6 | 1.44 | 0.47 | 4.43 |  |  |
| Dominant |  |  |  |  |  |  |  |  |  |
| T/T | 111 | 61.7 | 35 | 60.3 | 1 [Ref] |  |  | 0.8575 | 268.3 |
| C/T-C/C | 69 | 38.3 | 23 | 39.7 | 1.06 | 0.58 | 1.94 |  |  |
| Recessive |  |  |  |  |  |  |  |  |  |
| T/T-C/T | 169 | 93.9 | 53 | 91.4 | 1 [Ref] |  |  | 0.5179 | 267.9 |
| C/C | 11 | 6.1 | 5 | 8.6 | 1.45 | 0.48 | 4.36 |  |  |
| Overdominant |  |  |  |  |  |  |  |  |  |
| T/T-C/C | 122 | 67.8 | 40 | 69 | 1 [Ref] |  |  | 0.8658 | 268.3 |
| C/T | 58 | 32.2 | 18 | 31 | 0.95 | 0.5 | 1.79 |  |  |
| log-Additive |  |  |  |  |  |  |  |  |  |
| 0,1,2 | 180 | 75.6 | 58 | 24.4 | 1.1 | 0.69 | 1.77 | 0.6831 | 268.2 |
| SNP | Control | % | Case | % | OR | lower | upper | p-value | AIC |
| rs3744962 |  |  |  |  |  |  |  |  |  |
| Codominant |  |  |  |  |  |  |  |  |  |
| T/T | 164 | 92.1 | 55 | 72.4 | 1 [Ref] |  |  | - | 297.8 |
| C/T | 14 | 7.9 | 19 | 25 | 4.05 | 1.9 | 8.61 |  |  |
| C/C | 0 | 0 | 2 | 2.6 | 0 |  |  |  |  |
| Dominant |  |  |  |  |  |  |  |  |  |
| T/T | 164 | 92.1 | 55 | 72.4 | 1 [Ref] |  |  | 6.27E-05 | 298 |
| C/T-C/C | 14 | 7.9 | 21 | 27.6 | 4.47 | 2.13 | 9.39 |  |  |
| Recessive |  |  |  |  |  |  |  |  |  |
| T/T-C/T | 178 | 100 | 74 | 97.4 | 1 [Ref] |  |  | - | 309.1 |
| C/C | 0 | 0 | 2 | 2.6 | 0 |  |  |  |  |
| Overdominant |  |  |  |  |  |  |  |  |  |
| T/T-C/C | 164 | 92.1 | 57 | 75 | 1 [Ref] |  |  | 3.72E-04 | 301.3 |
| C/T | 14 | 7.9 | 19 | 25 | 3.9 | 1.84 | 8.29 |  |  |
| log-Additive |  |  |  |  |  |  |  |  |  |
| 0,1,2 | 178 | 70.1 | 76 | 29.9 | 4.36 | 2.13 | 8.93 | 4.83E-05 | 296.5 |
| SNP | Control | % | Case | % | OR | lower | upper | p-value | AIC |
| rs4075022 |  |  |  |  |  |  |  |  |  |
| Codominant |  |  |  |  |  |  |  |  |  |
| T/T | 106 | 59.2 | 26 | 39.4 | 1 [Ref] |  |  | 0.013212 | 282.8 |
| C/T | 59 | 33 | 29 | 43.9 | 2 | 1.08 | 3.72 |  |  |
| C/C | 14 | 7.8 | 11 | 16.7 | 3.2 | 1.3 | 7.87 |  |  |
| Dominant |  |  |  |  |  |  |  |  |  |
| T/T | 106 | 59.2 | 26 | 39.4 | 1 [Ref] |  |  | 0.005721 | 281.9 |
| C/T-C/C | 73 | 40.8 | 40 | 60.6 | 2.23 | 1.25 | 3.98 |  |  |
| Recessive |  |  |  |  |  |  |  |  |  |
| T/T-C/T | 165 | 92.2 | 55 | 83.3 | 1 [Ref] |  |  | 0.052054 | 285.7 |
| C/C | 14 | 7.8 | 11 | 16.7 | 2.36 | 1.01 | 5.5 |  |  |
| Overdominant |  |  |  |  |  |  |  |  |  |
| T/T-C/C | 120 | 67 | 37 | 56.1 | 1 [Ref] |  |  | 0.115007 | 287 |
| C/T | 59 | 33 | 29 | 43.9 | 1.59 | 0.89 | 2.84 |  |  |
| log-Additive |  |  |  |  |  |  |  |  |  |
| 0,1,2 | 179 | 73.1 | 66 | 26.9 | 1.85 | 1.22 | 2.79 | 0.003492 | 281 |
| SNP | Control | % | Case | % | OR | lower | upper | p-value | AIC |
| rs4075570 |  |  |  |  |  |  |  |  |  |
| Codominant |  |  |  |  |  |  |  |  |  |
| G/G | 57 | 31.8 | 13 | 19.1 | 1 [Ref] |  |  | 0.023586 | 289.2 |
| A/G | 82 | 45.8 | 29 | 42.6 | 1.55 | 0.74 | 3.24 |  |  |
| A/A | 40 | 22.3 | 26 | 38.2 | 2.85 | 1.31 | 6.21 |  |  |
| Dominant |  |  |  |  |  |  |  |  |  |
| G/G | 57 | 31.8 | 13 | 19.1 | 1 [Ref] |  |  | 0.041915 | 290.6 |
| A/G-A/A | 122 | 68.2 | 55 | 80.9 | 1.98 | 1 | 3.91 |  |  |
| Recessive |  |  |  |  |  |  |  |  |  |
| G/G-A/G | 139 | 77.7 | 42 | 61.8 | 1 [Ref] |  |  | 0.013597 | 288.6 |
| A/A | 40 | 22.3 | 26 | 38.2 | 2.15 | 1.18 | 3.93 |  |  |
| Overdominant |  |  |  |  |  |  |  |  |  |
| G/G-A/A | 97 | 54.2 | 39 | 57.4 | 1 [Ref] |  |  | 0.65497 | 294.5 |
| A/G | 82 | 45.8 | 29 | 42.6 | 0.88 | 0.5 | 1.55 |  |  |
| log-Additive |  |  |  |  |  |  |  |  |  |
| 0,1,2 | 179 | 72.5 | 68 | 27.5 | 1.7 | 1.15 | 2.51 | 0.006485 | 287.3 |
| SNP | Control | % | Case | % | OR | lower | upper | p-value | AIC |
| rs4245211 |  |  |  |  |  |  |  |  |  |
| Codominant |  |  |  |  |  |  |  |  |  |
| A/A | 71 | 41.5 | 32 | 43.2 | 1 [Ref] |  |  | 0.7887 | 305.7 |
| A/G | 81 | 47.4 | 32 | 43.2 | 0.88 | 0.49 | 1.57 |  |  |
| G/G | 19 | 11.1 | 10 | 13.5 | 1.17 | 0.49 | 2.79 |  |  |
| Dominant |  |  |  |  |  |  |  |  |  |
| A/A | 71 | 41.5 | 32 | 43.2 | 1 [Ref] |  |  | 0.8021 | 304.1 |
| A/G-G/G | 100 | 58.5 | 42 | 56.8 | 0.93 | 0.54 | 1.62 |  |  |
| Recessive |  |  |  |  |  |  |  |  |  |
| A/A-A/G | 152 | 88.9 | 64 | 86.5 | 1 [Ref] |  |  | 0.597 | 303.9 |
| G/G | 19 | 11.1 | 10 | 13.5 | 1.25 | 0.55 | 2.84 |  |  |
| Overdominant |  |  |  |  |  |  |  |  |  |
| A/A-G/G | 90 | 52.6 | 42 | 56.8 | 1 [Ref] |  |  | 0.5516 | 303.8 |
| A/G | 81 | 47.4 | 32 | 43.2 | 0.85 | 0.49 | 1.47 |  |  |
| log-Additive |  |  |  |  |  |  |  |  |  |
| 0,1,2 | 171 | 69.8 | 74 | 30.2 | 1.02 | 0.68 | 1.53 | 0.9418 | 304.2 |
| SNP | Control | % | Case | % | OR | lower | upper | p-value | AIC |
| rs4698951 |  |  |  |  |  |  |  |  |  |
| Codominant |  |  |  |  |  |  |  |  |  |
| T/T | 92 | 51.4 | 54 | 69.2 | 1 [Ref] |  |  | 0.002204 | 309.3 |
| C/T | 62 | 34.6 | 22 | 28.2 | 0.6 | 0.33 | 1.09 |  |  |
| C/C | 25 | 14 | 2 | 2.6 | 0.14 | 0.03 | 0.6 |  |  |
| Dominant |  |  |  |  |  |  |  |  |  |
| T/T | 92 | 51.4 | 54 | 69.2 | 1 [Ref] |  |  | 0.007292 | 312.3 |
| C/T-C/C | 87 | 48.6 | 24 | 30.8 | 0.47 | 0.27 | 0.83 |  |  |
| Recessive |  |  |  |  |  |  |  |  |  |
| T/T-C/T | 154 | 86 | 76 | 97.4 | 1 [Ref] |  |  | 0.002203 | 310.1 |
| C/C | 25 | 14 | 2 | 2.6 | 0.16 | 0.04 | 0.7 |  |  |
| Overdominant |  |  |  |  |  |  |  |  |  |
| T/T-C/C | 117 | 65.4 | 56 | 71.8 | 1 [Ref] |  |  | 0.308539 | 318.5 |
| C/T | 62 | 34.6 | 22 | 28.2 | 0.74 | 0.41 | 1.33 |  |  |
| log-Additive |  |  |  |  |  |  |  |  |  |
| 0,1,2 | 179 | 69.6 | 78 | 30.4 | 0.48 | 0.31 | 0.77 | 0.000953 | 308.6 |
| SNP | Control | % | Case | % | OR | lower | upper | p-value | AIC |
| rs566309 |  |  |  |  |  |  |  |  |  |
| Codominant |  |  |  |  |  |  |  |  |  |
| C/C | 153 | 85 | 60 | 78.9 | 1 [Ref] |  |  | - | 315.1 |
| C/T | 26 | 14.4 | 16 | 21.1 | 1.57 | 0.79 | 3.13 |  |  |
| T/T | 1 | 0.6 | 0 | 0 | 0 | 0 |  |  |  |
| Dominant |  |  |  |  |  |  |  |  |  |
| C/C | 153 | 85 | 60 | 78.9 | 1 [Ref] |  |  | 0.2445 | 314 |
| C/T-T/T | 27 | 15 | 16 | 21.1 | 1.51 | 0.76 | 3 |  |  |
| Recessive |  |  |  |  |  |  |  |  |  |
| C/C-C/T | 179 | 99.4 | 76 | 100 | 1 [Ref] |  |  | - | 314.7 |
| T/T | 1 | 0.6 | 0 | 0 | 0 | 0 |  |  |  |
| Overdominant |  |  |  |  |  |  |  |  |  |
| C/C-T/T | 154 | 85.6 | 60 | 78.9 | 1 [Ref] |  |  | 0.2004 | 313.8 |
| C/T | 26 | 14.4 | 16 | 21.1 | 1.58 | 0.79 | 3.15 |  |  |
| log-Additive |  |  |  |  |  |  |  |  |  |
| 0,1,2 | 180 | 70.3 | 76 | 29.7 | 1.42 | 0.73 | 2.76 | 0.4387 | 314.4 |
| SNP | Control | % | Case | % | OR | lower | upper | p-value | AIC |
| rs6507639 |  |  |  |  |  |  |  |  |  |
| Codominant |  |  |  |  |  |  |  |  |  |
| T/T | 155 | 86.1 | 64 | 86.5 | 1 [Ref] |  |  | - | 311.8 |
| G/T | 24 | 13.3 | 10 | 13.5 | 1.01 | 0.46 | 2.23 |  |  |
| G/G | 1 | 0.6 | 0 | 0 | 0 | 0 |  |  |  |
| Dominant |  |  |  |  |  |  |  |  |  |
| T/T | 155 | 86.1 | 64 | 86.5 | 1 [Ref] |  |  | 0.937 | 310.5 |
| G/T-G/G | 25 | 13.9 | 10 | 13.5 | 0.97 | 0.44 | 2.13 |  |  |
| Recessive |  |  |  |  |  |  |  |  |  |
| T/T-G/T | 179 | 99.4 | 74 | 100 | 1 [Ref] |  |  | - | 309.8 |
| G/G | 1 | 0.6 | 0 | 0 | 0 | 0 |  |  |  |
| Overdominant |  |  |  |  |  |  |  |  |  |
| T/T-G/G | 156 | 86.7 | 64 | 86.5 | 1 [Ref] |  |  | 0.9695 | 310.5 |
| G/T | 24 | 13.3 | 10 | 13.5 | 1.02 | 0.46 | 2.24 |  |  |
| log-Additive |  |  |  |  |  |  |  |  |  |
| 0,1,2 | 180 | 70.9 | 74 | 29.1 | 0.93 | 0.43 | 1.99 | - | 310.5 |
| SNP | Control | % | Case | % | OR | lower | upper | p-value | AIC |
| rs6578493 |  |  |  |  |  |  |  |  |  |
| Codominant |  |  |  |  |  |  |  |  |  |
| A/A | 114 | 64 | 45 | 63.4 | 1 [Ref] |  |  | 0.575 | 302.6 |
| A/C | 59 | 33.1 | 22 | 31 | 0.94 | 0.52 | 1.72 |  |  |
| C/C | 5 | 2.8 | 4 | 5.6 | 2.03 | 0.52 | 7.89 |  |  |
| Dominant |  |  |  |  |  |  |  |  |  |
| A/A | 114 | 64 | 45 | 63.4 | 1 [Ref] |  |  | 0.9215 | 301.7 |
| A/C-C/C | 64 | 36 | 26 | 36.6 | 1.03 | 0.58 | 1.82 |  |  |
| Recessive |  |  |  |  |  |  |  |  |  |
| A/A-A/C | 173 | 97.2 | 67 | 94.4 | 1 [Ref] |  |  | 0.3005 | 300.6 |
| C/C | 5 | 2.8 | 4 | 5.6 | 2.07 | 0.54 | 7.93 |  |  |
| Overdominant |  |  |  |  |  |  |  |  |  |
| A/A-C/C | 119 | 66.9 | 49 | 69 | 1 [Ref] |  |  | 0.7419 | 301.6 |
| A/C | 59 | 33.1 | 22 | 31 | 0.91 | 0.5 | 1.64 |  |  |
| log-Additive |  |  |  |  |  |  |  |  |  |
| 0,1,2 | 178 | 71.5 | 71 | 28.5 | 1.12 | 0.69 | 1.82 | 0.6576 | 301.5 |
| SNP | Control | % | Case | % | OR | lower | upper | p-value | AIC |
| rs664677 |  |  |  |  |  |  |  |  |  |
| Codominant |  |  |  |  |  |  |  |  |  |
| T/T | 70 | 39.1 | 33 | 47.1 | 1 [Ref] |  |  | 0.5133 | 300.5 |
| C/T | 85 | 47.5 | 29 | 41.4 | 0.72 | 0.4 | 1.31 |  |  |
| C/C | 24 | 13.4 | 8 | 11.4 | 0.71 | 0.29 | 1.74 |  |  |
| Dominant |  |  |  |  |  |  |  |  |  |
| T/T | 70 | 39.1 | 33 | 47.1 | 1 [Ref] |  |  | 0.2486 | 298.5 |
| C/T-C/C | 109 | 60.9 | 37 | 52.9 | 0.72 | 0.41 | 1.26 |  |  |
| Recessive |  |  |  |  |  |  |  |  |  |
| T/T-C/T | 155 | 86.6 | 62 | 88.6 | 1 [Ref] |  |  | 0.6717 | 299.6 |
| C/C | 24 | 13.4 | 8 | 11.4 | 0.83 | 0.36 | 1.95 |  |  |
| Overdominant |  |  |  |  |  |  |  |  |  |
| T/T-C/C | 94 | 52.5 | 41 | 58.6 | 1 [Ref] |  |  | 0.3875 | 299.1 |
| C/T | 85 | 47.5 | 29 | 41.4 | 0.78 | 0.45 | 1.37 |  |  |
| log-Additive |  |  |  |  |  |  |  |  |  |
| 0,1,2 | 179 | 71.9 | 70 | 28.1 | 0.8 | 0.53 | 1.21 | 0.2926 | 298.7 |
| SNP | Control | % | Case | % | OR | lower | upper | p-value | AIC |
| rs6825379 | Control | % | Case | % | OR | lower | upper | p-value | AI |
| Codominant |  |  |  |  |  |  |  |  |  |
| A/A | 132 | 73.7 | 53 | 72.6 | 1 [Ref] |  |  | - | 304.8 |
| A/G | 41 | 22.9 | 20 | 27.4 | 1.21 | 0.65 | 2.26 |  |  |
| G/G | 6 | 3.4 | 0 | 0 | 0 | 0 |  |  |  |
| Dominant |  |  |  |  |  |  |  |  |  |
| A/A | 132 | 73.7 | 53 | 72.6 | 1 [Ref] |  |  | 0.8528 | 307.3 |
| A/G-G/G | 47 | 26.3 | 20 | 27.4 | 1.06 | 0.57 | 1.96 |  |  |
| Recessive |  |  |  |  |  |  |  |  |  |
| A/A-A/G | 173 | 96.6 | 73 | 100 | 1 [Ref] |  |  | - | 303.2 |
| G/G | 6 | 3.4 | 0 | 0 | 0 | 0 |  |  |  |
| Overdominant |  |  |  |  |  |  |  |  |  |
| A/A-G/G | 138 | 77.1 | 53 | 72.6 | 1 [Ref] |  |  | 0.4538 | 306.8 |
| A/G | 41 | 22.9 | 20 | 27.4 | 1.27 | 0.68 | 2.36 |  |  |
| log-Additive |  |  |  |  |  |  |  |  |  |
| 0,1,2 | 179 | 71 | 73 | 29 | 0.92 | 0.53 | 1.58 | 0.2749 | 307.2 |
| SNP | Control | % | Case | % | OR | lower | upper | p-value | AIC |
| rs6983267 |  |  |  |  |  |  |  |  |  |
| Codominant |  |  |  |  |  |  |  |  |  |
| G/G | 104 | 57.8 | 19 | 30.6 | 1 [Ref] |  |  | 6.37E-05 | 262.1 |
| G/T | 51 | 28.3 | 37 | 59.7 | 3.97 | 2.08 | 7.58 |  |  |
| T/T | 25 | 13.9 | 6 | 9.7 | 1.31 | 0.48 | 3.63 |  |  |
| Dominant |  |  |  |  |  |  |  |  |  |
| G/G | 104 | 57.8 | 19 | 30.6 | 1 [Ref] |  |  | 1.99E-04 | 265.6 |
| G/T-T/T | 76 | 42.2 | 43 | 69.4 | 3.1 | 1.67 | 5.73 |  |  |
| Recessive |  |  |  |  |  |  |  |  |  |
| G/G-G/T | 155 | 86.1 | 56 | 90.3 | 1 [Ref] |  |  | 3.80E-01 | 278.6 |
| T/T | 25 | 13.9 | 6 | 9.7 | 0.66 | 0.26 | 1.7 |  |  |
| Overdominant |  |  |  |  |  |  |  |  |  |
| G/G-T/T | 129 | 71.7 | 25 | 40.3 | 1 [Ref] |  |  | 1.27E-05 | 260.4 |
| G/T | 51 | 28.3 | 37 | 59.7 | 3.74 | 2.05 | 6.84 |  |  |
| log-Additive |  |  |  |  |  |  |  |  |  |
| 0,1,2 | 180 | 74.4 | 62 | 25.6 | 1.57 | 1.05 | 2.35 | 2.84E-02 | 274.6 |
| SNP | Control | % | Case | % | OR | lower | upper | p-value | AIC |
| rs7028661 |  |  |  |  |  |  |  |  |  |
| Codominant |  |  |  |  |  |  |  |  |  |
| G/G | 92 | 51.1 | 30 | 42.9 | 1 [Ref] |  |  | 0.4851 | 301 |
| A/G | 70 | 38.9 | 31 | 44.3 | 1.36 | 0.75 | 2.45 |  |  |
| A/A | 18 | 10 | 9 | 12.9 | 1.53 | 0.62 | 3.77 |  |  |
| Dominant |  |  |  |  |  |  |  |  |  |
| G/G | 92 | 51.1 | 30 | 42.9 | 1 [Ref] |  |  | 0.2404 | 299.1 |
| A/G-A/A | 88 | 48.9 | 40 | 57.1 | 1.39 | 0.8 | 2.43 |  |  |
| Recessive |  |  |  |  |  |  |  |  |  |
| G/G-A/G | 162 | 90 | 61 | 87.1 | 1 [Ref] |  |  | 0.5199 | 300.1 |
| A/A | 18 | 10 | 9 | 12.9 | 1.33 | 0.57 | 3.11 |  |  |
| Overdominant |  |  |  |  |  |  |  |  |  |
| G/G-A/A | 110 | 61.1 | 39 | 55.7 | 1 [Ref] |  |  | 0.4362 | 299.9 |
| A/G | 70 | 38.9 | 31 | 44.3 | 1.25 | 0.71 | 2.18 |  |  |
| log-Additive |  |  |  |  |  |  |  |  |  |
| 0,1,2 | 180 | 72 | 70 | 28 | 1.27 | 0.85 | 1.91 | 0.2434 | 299.1 |
| SNP | Control | % | Case | % | OR | lower | upper | p-value | AIC |
| rs7037324 |  |  |  |  |  |  |  |  |  |
| Codominant |  |  |  |  |  |  |  |  |  |
| G/G | 107 | 59.8 | 35 | 48.6 | 1 [Ref] |  |  | 0.2332 | 303.9 |
| A/G | 55 | 30.7 | 30 | 41.7 | 1.67 | 0.93 | 3 |  |  |
| A/A | 17 | 9.5 | 7 | 9.7 | 1.26 | 0.48 | 3.29 |  |  |
| Dominant |  |  |  |  |  |  |  |  |  |
| G/G | 107 | 59.8 | 35 | 48.6 | 1 [Ref] |  |  | 0.1074 | 302.3 |
| A/G-A/A | 72 | 40.2 | 37 | 51.4 | 1.57 | 0.91 | 2.72 |  |  |
| Recessive |  |  |  |  |  |  |  |  |  |
| G/G-A/G | 162 | 90.5 | 65 | 90.3 | 1 [Ref] |  |  | 0.9563 | 304.9 |
| A/A | 17 | 9.5 | 7 | 9.7 | 1.03 | 0.41 | 2.59 |  |  |
| Overdominant |  |  |  |  |  |  |  |  |  |
| G/G-A/A | 124 | 69.3 | 42 | 58.3 | 1 [Ref] |  |  | 0.1006 | 302.2 |
| A/G | 55 | 30.7 | 30 | 41.7 | 1.61 | 0.91 | 2.84 |  |  |
| log-Additive |  |  |  |  |  |  |  |  |  |
| 0,1,2 | 179 | 71.3 | 72 | 28.7 | 1.29 | 0.86 | 1.93 | 0.2228 | 303.4 |
| SNP | Control | % | Case | % | OR | lower | upper | p-value | AIC |
| rs944289 |  |  |  |  |  |  |  |  |  |
| Codominant |  |  |  |  |  |  |  |  |  |
| C/C | 88 | 48.9 | 26 | 33.8 | 1 [Ref] |  |  | 0.00759 | 310.1 |
| C/T | 52 | 28.9 | 38 | 49.4 | 2.47 | 1.35 | 4.53 |  |  |
| T/T | 40 | 22.2 | 13 | 16.9 | 1.1 | 0.51 | 2.36 |  |  |
| Dominant |  |  |  |  |  |  |  |  |  |
| C/C | 88 | 48.9 | 26 | 33.8 | 1 [Ref] |  |  | 0.02427 | 312.7 |
| C/T-T/T | 92 | 51.1 | 51 | 66.2 | 1.88 | 1.08 | 3.27 |  |  |
| Recessive |  |  |  |  |  |  |  |  |  |
| C/C-C/T | 140 | 77.8 | 64 | 83.1 | 1 [Ref] |  |  | 0.32551 | 316.8 |
| T/T | 40 | 22.2 | 13 | 16.9 | 0.71 | 0.36 | 1.42 |  |  |
| Overdominant |  |  |  |  |  |  |  |  |  |
| C/C-T/T | 128 | 71.1 | 39 | 50.6 | 1 [Ref] |  |  | 0.00184 | 308.1 |
| C/T | 52 | 28.9 | 38 | 49.4 | 2.4 | 1.38 | 4.16 |  |  |
| log-Additive |  |  |  |  |  |  |  |  |  |
| 0,1,2 | 180 | 70 | 77 | 30 | 1.18 | 0.83 | 1.66 | 0.35246 | 317 |
| SNP | Control | % | Case | % | OR | lower | upper | p-value | AIC |
| rs949908 |  |  |  |  |  |  |  |  |  |
| Codominant |  |  |  |  |  |  |  |  |  |
| T/T | 55 | 32.2 | 18 | 24.3 | 1 [Ref] |  |  | 0.2848 | 303.7 |
| A/T | 72 | 42.1 | 39 | 52.7 | 1.66 | 0.86 | 3.2 |  |  |
| A/A | 44 | 25.7 | 17 | 23 | 1.18 | 0.55 | 2.56 |  |  |
| Dominant |  |  |  |  |  |  |  |  |  |
| T/T | 55 | 32.2 | 18 | 24.3 | 1 [Ref] |  |  | 0.2127 | 302.6 |
| A/T-A/A | 116 | 67.8 | 56 | 75.7 | 1.48 | 0.79 | 2.74 |  |  |
| Recessive |  |  |  |  |  |  |  |  |  |
| T/T-A/T | 127 | 74.3 | 57 | 77 | 1 [Ref] |  |  | 0.645 | 304 |
| A/A | 44 | 25.7 | 17 | 23 | 0.86 | 0.45 | 1.63 |  |  |
| Overdominant |  |  |  |  |  |  |  |  |  |
| T/T-A/A | 99 | 57.9 | 35 | 47.3 | 1 [Ref] |  |  | 0.1265 | 301.8 |
| A/T | 72 | 42.1 | 39 | 52.7 | 1.53 | 0.89 | 2.65 |  |  |
| log-Additive |  |  |  |  |  |  |  |  |  |
| 0,1,2 | 171 | 69.8 | 74 | 30.2 | 1.1 | 0.76 | 1.59 | 0.6207 | 303.9 |
| SNP | Control | % | Case | % | OR | lower | upper | p-value | AIC |
| rs965513 |  |  |  |  |  |  |  |  |  |
| Codominant |  |  |  |  |  |  |  |  |  |
| G/G | 99 | 55 | 34 | 44.7 | 1 [Ref] |  |  | 0.3232 | 315.1 |
| A/G | 64 | 35.6 | 33 | 43.4 | 1.5 | 0.85 | 2.66 |  |  |
| A/A | 17 | 9.4 | 9 | 11.8 | 1.54 | 0.63 | 3.78 |  |  |
| Dominant |  |  |  |  |  |  |  |  |  |
| G/G | 99 | 55 | 34 | 44.7 | 1 [Ref] |  |  | 0.1331 | 313.1 |
| A/G-A/A | 81 | 45 | 42 | 55.3 | 1.51 | 0.88 | 2.59 |  |  |
| Recessive |  |  |  |  |  |  |  |  |  |
| G/G-A/G | 163 | 90.6 | 67 | 88.2 | 1 [Ref] |  |  | 0.5668 | 315.1 |
| A/A | 17 | 9.4 | 9 | 11.8 | 1.29 | 0.55 | 3.03 |  |  |
| Overdominant |  |  |  |  |  |  |  |  |  |
| G/G-A/A | 116 | 64.4 | 43 | 56.6 | 1 [Ref] |  |  | 0.238 | 314 |
| A/G | 64 | 35.6 | 33 | 43.4 | 1.39 | 0.81 | 2.4 |  |  |
| log-Additive |  |  |  |  |  |  |  |  |  |
| 0,1,2 | 180 | 70.3 | 76 | 29.7 | 1.32 | 0.89 | 1.96 | 0.1691 | 313.5 |
| SNP | Control | % | Case | % | OR | lower | upper | p-value | AIC |
| rs9937860 |  |  |  |  |  |  |  |  |  |
| Codominant |  |  |  |  |  |  |  |  |  |
| T/T | 137 | 77.8 | 52 | 69.3 | 1 [Ref] |  |  | 0.3664 | 310.1 |
| G/T | 30 | 17 | 18 | 24 | 1.58 | 0.81 | 3.08 |  |  |
| G/G | 9 | 5.1 | 5 | 6.7 | 1.46 | 0.47 | 4.57 |  |  |
| Dominant |  |  |  |  |  |  |  |  |  |
| T/T | 137 | 77.8 | 52 | 69.3 | 1 [Ref] |  |  | 0.158 | 308.2 |
| G/T-G/G | 39 | 22.2 | 23 | 30.7 | 1.55 | 0.85 | 2.85 |  |  |
| Recessive |  |  |  |  |  |  |  |  |  |
| T/T-G/T | 167 | 94.9 | 70 | 93.3 | 1 [Ref] |  |  | 0.629 | 309.9 |
| G/G | 9 | 5.1 | 5 | 6.7 | 1.33 | 0.43 | 4.1 |  |  |
| Overdominant |  |  |  |  |  |  |  |  |  |
| T/T-G/G | 146 | 83 | 57 | 76 | 1 [Ref] |  |  | 0.2069 | 308.6 |
| G/T | 30 | 17 | 18 | 24 | 1.54 | 0.79 | 2.97 |  |  |
| log-Additive |  |  |  |  |  |  |  |  |  |
| 0,1,2 | 176 | 70.1 | 75 | 29.9 | 1.35 | 0.85 | 2.12 | 0.2067 | 308.5 |
| SNP | Control | % | Case | % | OR | lower | upper | p-value | AIC |
| rs9943744 |  |  |  |  |  |  |  |  |  |
| Codominant |  |  |  |  |  |  |  |  |  |
| C/C | 60 | 33.7 | 15 | 19.7 | 1 [Ref] |  |  | 0.008327 | 306.4 |
| C/T | 86 | 48.3 | 35 | 46.1 | 1.63 | 0.82 | 3.24 |  |  |
| T/T | 32 | 18 | 26 | 34.2 | 3.25 | 1.51 | 7 |  |  |
| Dominant |  |  |  |  |  |  |  |  |  |
| C/C | 60 | 33.7 | 15 | 19.7 | 1 [Ref] |  |  | 0.022034 | 308.7 |
| C/T-T/T | 118 | 66.3 | 61 | 80.3 | 2.07 | 1.09 | 3.94 |  |  |
| Recessive |  |  |  |  |  |  |  |  |  |
| C/C-C/T | 146 | 82 | 50 | 65.8 | 1 [Ref] |  |  | 0.005867 | 306.4 |
| T/T | 32 | 18 | 26 | 34.2 | 2.37 | 1.29 | 4.36 |  |  |
| Overdominant |  |  |  |  |  |  |  |  |  |
| C/C-T/T | 92 | 51.7 | 41 | 53.9 | 1 [Ref] |  |  | 0.740916 | 313.9 |
| C/T | 86 | 48.3 | 35 | 46.1 | 0.91 | 0.53 | 1.56 |  |  |
| log-Additive |  |  |  |  |  |  |  |  |  |
| 0,1,2 | 178 | 70.1 | 76 | 29.9 | 1.81 | 1.23 | 2.67 | 0.002119 | 304.5 |
| SNP | Control | % | Case | % | OR | lower | upper | p-value | AIC |
| rs9993140 |  |  |  |  |  |  |  |  |  |
| Codominant |  |  |  |  |  |  |  |  |  |
| G/G | 161 | 89.4 | 74 | 100 | 1 [Ref] |  |  | - | 298.8 |
| A/G | 18 | 10 | 0 | 0 | 0 | 0 |  |  |  |
| A/A | 1 | 0.6 | 0 | 0 | 0 | 0 |  |  |  |
| Dominant |  |  |  |  |  |  |  |  |  |
| G/G | 161 | 89.4 | 74 | 100 | 1 [Ref] |  |  | - | 296.8 |
| A/G-A/A | 19 | 10.6 | 0 | 0 | 0 | 0 |  |  |  |
| Recessive |  |  |  |  |  |  |  |  |  |
| G/G-A/G | 179 | 99.4 | 74 | 100 | 1 [Ref] |  |  | - | 309.8 |
| A/A | 1 | 0.6 | 0 | 0 | 0 | 0 |  |  |  |
| Overdominant |  |  |  |  |  |  |  |  |  |
| G/G-A/A | 162 | 90 | 74 | 100 | 1 [Ref] |  |  | - | 297.5 |
| A/G | 18 | 10 | 0 | 0 | 0 | 0 |  |  |  |
| log-Additive |  |  |  |  |  |  |  |  |  |
| 0,1,2 | 180 | 70.9 | 74 | 29.1 | 0 | 0 |  | - | 296.8 |
